# The Gondwana Breakup and the History of the Atlantic and Indian Oceans Unveils Two New Clades for Early Neobatrachian Diversification

**DOI:** 10.1371/journal.pone.0143926

**Published:** 2015-11-30

**Authors:** Annelise Frazão, Hélio Ricardo da Silva, Claudia Augusta de Moraes Russo

**Affiliations:** 1 Departamento de Genética, Instituto de Biologia, Universidade Federal do Rio de Janeiro, Rio de Janeiro, Rio de Janeiro, Brazil; 2 Departamento de Biologia Animal, Instituto de Biologia, Universidade Federal Rural do Rio de Janeiro, Seropédica, Rio de Janeiro, Brazil; University of Chicago, UNITED STATES

## Abstract

The largest anuran diversity belongs to the Neobatrachia, which harbor more than five thousand extant species. Here, we propose a new hypothesis for the historical aspects of the neobatrachian evolution with a formal biogeographical analysis. We selected 12 genes for 144 neobatrachian genera and four archaeobatrachian outgroups and performed a phylogenetic analysis using a maximum likelihood algorithm with the rapid bootstrap test. We also estimated divergence times for major lineages using a relaxed uncorrelated clock method. According to our time scale, the diversification of crown Neobatrachia began around the end of the Early Cretaceous. Our phylogenetic tree suggests that the first split of Neobatrachia is related to the geological events in the Atlantic and Indian Oceans. Hence, we propose names for these clades that indicate this connection, i.e., Atlanticanura and Indianura. The Atlanticanura is composed of three major neobatrachian lineages: Heleophrynidae, Australobatrachia and Nobleobatrachia. On the other hand, the Indianura consists of two major lineages: Sooglossoidea and Ranoides. The biogeographical analysis indicates that many neobatrachian splits occurred as a result of geological events such as the separation between South America and Africa, between India and the Seychelles, and between Australia and South America.

## Introduction

Frogs and toads are members of the Anura clade, which inhabits forests, savannas, and even some deserts across the globe. The clade is currently divided into two groups, the paraphyletic Archaeaobatrachia and the Neobatrachia [1]. The first group includes the model African clawed toad *Xenopus laevis* and other lineages with a unique combination of characteristics, such as ribs that are absent in most other frogs. The largest anuran diversity, however, belongs to the Neobatrachia, which harbors more than five thousand extant species [2].

The oldest neobatrachian fossil records were found in northern South America in mid-Cretaceous sediments [3]. These records suggest a Gondwanan origin and a link between the supercontinent breakup and the early patterns of the group’s diversification [4,5]. The five major neobatrachian lineages, Sooglossoidea, Australobatrachia, Heleophrynidae, Ranoides, and Nobleobatrachia [2,6], fit well into the Gondwana hypothesis.

Recent studies, for instance, indicate that the Indian Nasikabatrachidae is closely related to the Seychellois Sooglossidae [7]. The morphologically odd members of the lineage comprised by these families inhabit two landmasses that have a close geological affinity with Gondwana [8]. The second lineage is known as the Australobatrachia, whose geographical distribution also relates to the supercontinent, as it includes the Chilean family Calyptocephalellidae [9] and the Australian families Limnodynastidae and Myobatrachidae. The third lineage is the small family Heleophrynidae from South Africa. Finally, the Nobleobatrachia and Ranoides lineages are cosmopolitan, including the vast majority of neobatrachian diversity found on the planet [2]. Despite the fact that the monophyletic status of these five lineages is well established, the historical aspects that promoted their diversification in Gondwana remain controversial [3]. As the fossil record is more copious in the recent time scale [4], molecular data is the best way to understand the history of neobatrachian diversification.

Nonetheless, molecular studies have reported mixed results for the main phylogenetic arrangements in the group. Roelants and co-workers [4], for instance, gathered a large molecular dataset with 171 species and their tree topology showed Heleophrynidae as the sister group of the other four lineages grouped in pairs: Australobatrachia plus Nobleobatrachia and Sooglossoidea (Nasikabatrachidae and Sooglossidae) plus Ranoides (see also [5]). Nevertheless, those authors were concerned with the phylogenetic patterns not in terms of the biogeographical implications or the association with the breakup of Gondwanaland. With a more biogeographical focus, the study by Biju and Bossuyt [7] recovered Sooglossoidea as the sister group of the remaining neobatrachians. That study, however, did not include all neobatrachian lineages, as Calyptocephalellidae that was not included in their dataset.

A taxon sampling exception was the comprehensive study by Pyron and Wiens [10,11], in which thousands of species from the five neobatrachian lineages were analyzed (see also [11]). Their study indicates Heleophrynidae as the sister group of all other neobatrachians, but with Sooglossoidea diverging afterwards as sister to the other three lineages. In that study, however, the amount of missing data in the alignment was quite large (around 70%), which has been considered phylogenetically worrisome [12].

Herein, we propose a new hypothesis for the diversification of the Neobatrachia and its association with the breakup of the supercontinent Gondwana. For this purpose, we have estimated a time-tree and performed a formal biogeographic analysis using a large dataset of 12 loci and 144 neobatrachian genera, assuming the monophyletic status of genera to increase matrix-filling levels.

## Material and Methods

### Taxonomic reference and molecular data

In this work, our taxonomic reference was the on-line database Amphibian Species of the World, version 6.0, which is maintained by the American Museum of Natural History (ASW) [13]. This curated database contains nominal amphibian species as well as their classification into higher taxonomical groups.

The search for amphibian species sequences started with a preliminary exploration on the on-line database Phylota [14], which assembles nucleotide sequences from the GenBank by taxonomic names [15]. In this preliminary search, we selected 12 genes, including nuclear and mitochondrial loci. The nuclear genes selected were: *c-myc* (myelocytomatosis exon 1 and 2), *H3A* (histone 3a), *POMC* (proopiomelanocortin), *RAG*-1 (recombination activating gene 1), *RHOD* (rhodopsin), *SIA* (seventh in absentia), *TyrPrecur* (tyrodinase precursor), and ribosomal 28S. We also included four mitochondrial genes: *cyt*-b (cytochrome b), *ND-*1 (NADH dehydrogenase subunit 1) and the ribosomal 12S and 16S genes.

As previously mentioned, in order to maximize alignment matrix filling levels, we assumed generic monophyly. Our final dataset included 144 neobatrachian genera and four archaeobatrachian outgroups: *Bombina* (Bombinatoridae), *Pelobates* (Pelobatidae), *Pipa* and *Xenopus* (Pipidae). For most neobatrachian families, up to five genera were selected per family in order to include a wide geographical distribution. This was not done for four families: Bufonidae, Microhylidae, Limnodynastidae and Myobatrachidae. In Bufonidae, we sampled species that were representative of all genera in order to better cover the wide geographical distribution of the family members. Conversely, for Microhylidae, a single genus for each of the subfamilies was sampled to include major lineages. Finally, for Myobatrachoidea, all genera with available sequences were included due to the questionable monophyletic status of the families [2,10]. GenBank accession numbers for all sequences are listed in S1 Table.

### Alignment and phylogenetic inference

Alignments were conducted separately for each marker using the Q-INS-I option for ribosomal markers and automatic option for remaining markers implemented in the MAFFT on-line algorithm, version 6.0 [16]. Gaps, missing and ambiguous data were eliminated using the default parameters of the Gblocks program [17]. Individual gene alignments were concatenated using SEAVIEW [18]. The final alignment contained 8,145 bp (55% filled matrix) and is available for download at Figshare repository (http://dx.doi.org/10.6084/m9.figshare.1538630).

We performed a phylogenetic analysis using the maximum likelihood (ML) method. In order to select the ML final tree, the rapid bootstrap algorithm (RBS) was implemented, using 1,000 non-parametric bootstrap replicates and the thorough ML search option [19]. The ML analysis and the bootstrap test were executed using the RAxML program, version 7.3.2, available on-line through the CIPRES project [20]. We used the CAT approximation for rate heterogeneity using per-site corrections, as it requires less computational time than the GAMMA method [21]. Furthermore, some authors have suggested that GTRGAMMA should be used without invariant sites [22]. The PartitionFinder program [23] indicated the best-fitting partition scheme for our dataset. The final partition scheme is available in S1 File.

In order to determine the influence of outgroups in our topology, we assembled an alignment with no outgroups for the 12 markers. The resulting tree shows that our two new clades (Indianura and Atlanticanura) are recovered with maximum bootstrap support (S1 Fig). This result provides support for our diversification hypothesis and suggests that long branch attraction may be lowering the bootstrap values for these clades in the analysis with outgroups.

### Divergence time estimation

The divergence time estimation was inferred with a Bayesian method using BEAST, version 1.7.5 [24], also available on the CIPRES web server [20]. The data method implemented in this program assumes a relaxed uncorrelated clock in which the rate for each branch is drawn independently from an underlying lognormal distribution [25]. The partition scheme and the evolutionary model were selected using PartitionFinder [23] (see S1 File). Cladogenesis was described using the Yule Process. Our final ML tree was included as starting tree and this topology was not allowed to vary during Markov Chain Monte Carlo (MCMC) sampling.

In BEAST, the six calibration points used as time priors require a distribution. In all cases, the normal distribution was assumed. This is recommended because the exact positions of the fossils on our tree were somewhat dubious. This type of distribution is considered a conservative option due to the soft bounds at the maximum and minimum ages [26].

**Fig 1:**
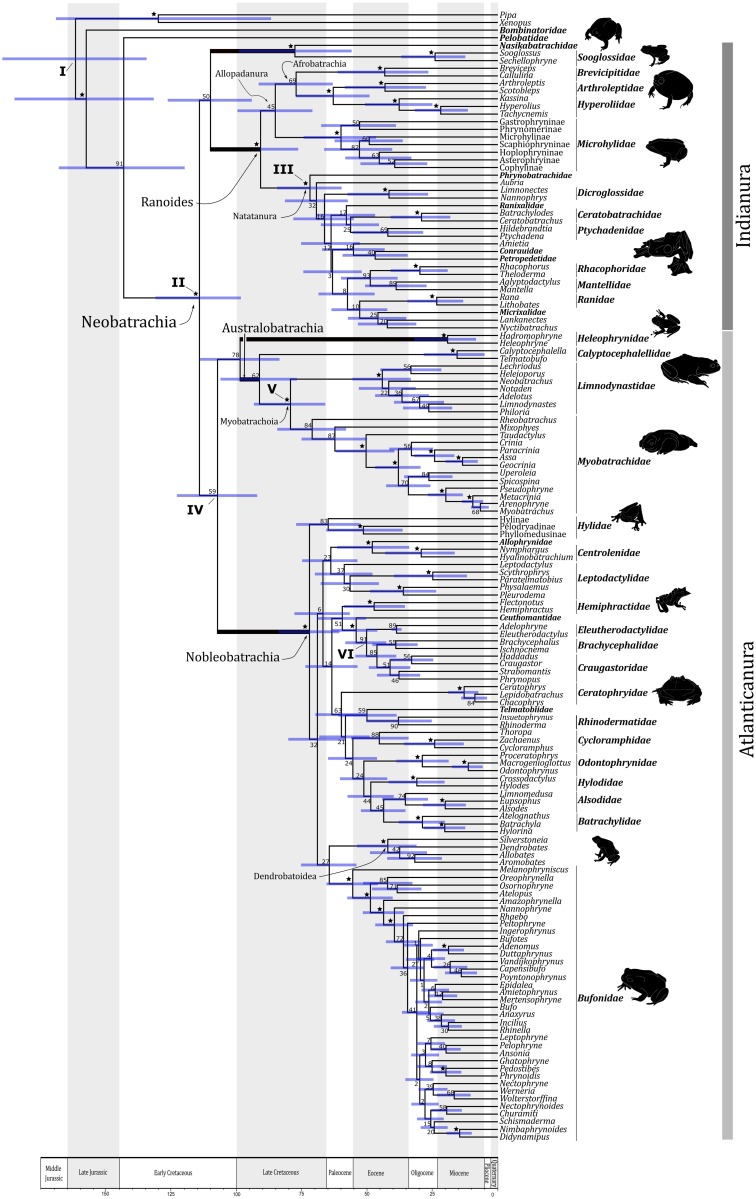
Bayesian time-scale for Neobatrachia. Chronogram for Neobatrachia derived from Bayesian analysis employing a relaxed molecular clock. Stars next nodes indicate the bootstrap value ≥ 95. Bootstrap values lower than 95 are shown next to corresponding nodes. The horizontal blue bars represents 95% of highest posterior density (HPD). The highlight branches (bold branches) represent the five major lineages of Neobatrachia. The Roman numbers at the nodes mark the position of the fossil calibrations. The time scale measures time in millions of years.

All calibration points were chosen based on the on-line resource Lisanfos KMS, version 1.2 [9], which compiles amphibian fossil data. Using a conservative criterion, we have selected only the records with an at least partially complete skeleton and a detailed fossil description for the time analysis. Details on fossil dates and prior distribution parameters for each calibration point are provided in S2 File and S2 Fig. Using these fossils, we constrained the dates for the following nodes (see Fig 1):

1. The split of Xenoanura and Sokolanura (root calibration): 145 Ma as minimum age for *Rhadinosteus parvus* [27] and 251 Ma as maximum age for *Triadobatrachus massinoti* [28].
2. The split of Neobatrachia crown group: 99.6 Ma as minimum age for *Cratia gracilis* [3] and 161.2 Ma as maximum age for *Rhadinosteus parvus* [27].
3. The split of Natatanura crown group: 34 Ma as minimum age for *Thaumastosaurus gezei* [29] and 125 Ma as maximum age for *Arariphrynus placidoi* [30].
4. The split of Nobleobatrachia stem group: 83.5 Ma as minimum age for *Baurubatrachus pricei* [31] and 125 Ma as maximum age for *Eurycaphalella alcinae* [3].
5. The split of Myobatrachidae and Limnodynastidae: 65.5 Ma as minimum age for *Indobatrachus pisillus* [32] and 125 Ma as maximum age for *Cratia gracilis* [3].
6. The split of Eleutherodactylidae stem group: 34 Ma as minimum age and 40 Ma as maximum age for *Eleutherodactylis* sp. [33].

The MCMC search included 10 independent runs of 100 million generations each, sampling every 10,000 generations. In each run, the first one million generations were discarded as burn-in. Convergence and stationary levels were checked with TRACER version 1.5 [34] until the effective sample size (ESS) scores were greater than 200, as recommended by Rambaut and Drummond [34]. The results from the 10 independent runs were combined using the BEAST package, TreeAnnotator version 1.7.5, and visualized using FigTree, version 1.4 [35].

### Biogeographic Analysis

The biogeographic areas are delimited in S2 File (see also [36]). The current distribution of neobatrachians was defined according to ASW [13] (see S2 Table). For this analysis, we used the Dispersal-Extinction-Cladogenesis (DEC) model of range evolution implemented in a likelihood framework [37] (Ree and Smith, 2008) in the Lagrange software [38]. In particular, the DEC model uses a time-scale to estimate the probability of change between geographical areas given the branch length and the current geographical distribution of taxa [37–39].

The mean age for the root node, as estimated in our divergence time analysis, was set as root of phylogeny for the Lagrange analysis. We conducted two Lagrange analyses: the first included five time slices ranging from 150 million years ago up to the present, each with a time span of 30 million years. The DEC model may incorporate information on past geological events, specifying an instantaneous transition probability between geographical ranges [37–39]. Given the transition matrix [37], the probability of geographical range changes along the branches of the time-tree is calculated for each constrained time slice.

The method implements the so-called reticulate model, in which geographical areas undergo cyclic events of area connections and splitting over time [40]. Thus, the matrix of instantaneous transition rates between areas change according to the time slice being considered. For instance, we established the value of 1.0 for dispersal between connected areas, of 0.5 for connections by islands and of 0.01 for all other cases. The time slice information with the respective matrix of connection possibilities is available in S2 File.

The second run was performed with no time constraints in order to verify whether these would influence our results. A significance test was performed comparing the global likelihoods between these runs. If one value is larger than the other by two log-likelihood units, they are considered significantly different [38]. Indeed, the results of this analysis showed that the model using a constrained adjacency matrix (-lnL = 271.5) was significantly more likely than the model using an unconstrained adjacency matrix (-lnL = 282.2). This result strongly indicates that our time slices provided valuable information regarding the biogeographical history of Neobatrachia. Geographical ancestral areas were assumed to include up to three areas.

## Results

The phylogenetic tree resulting from the likelihood analysis (Fig 1) recovers Neobatrachia (Bootstrap Proportion—BP100) as being closely related (91 BP) to the Pelobatidae family, which is part of the Anomocoela clade [2,4–5,10,41–43]. Nevertheless, many nodes in our topology show low bootstrap proportion values. For this reason, previous topologies [2,4,10] are not significantly rejected by our analysis indicating that further information is needed to disclose a more conclusive phylogenetic scenario for the group (Fig 1). The maximum likelihood tree recovered here showing the branch length information is available in S2 Fig.

**Fig 2:**
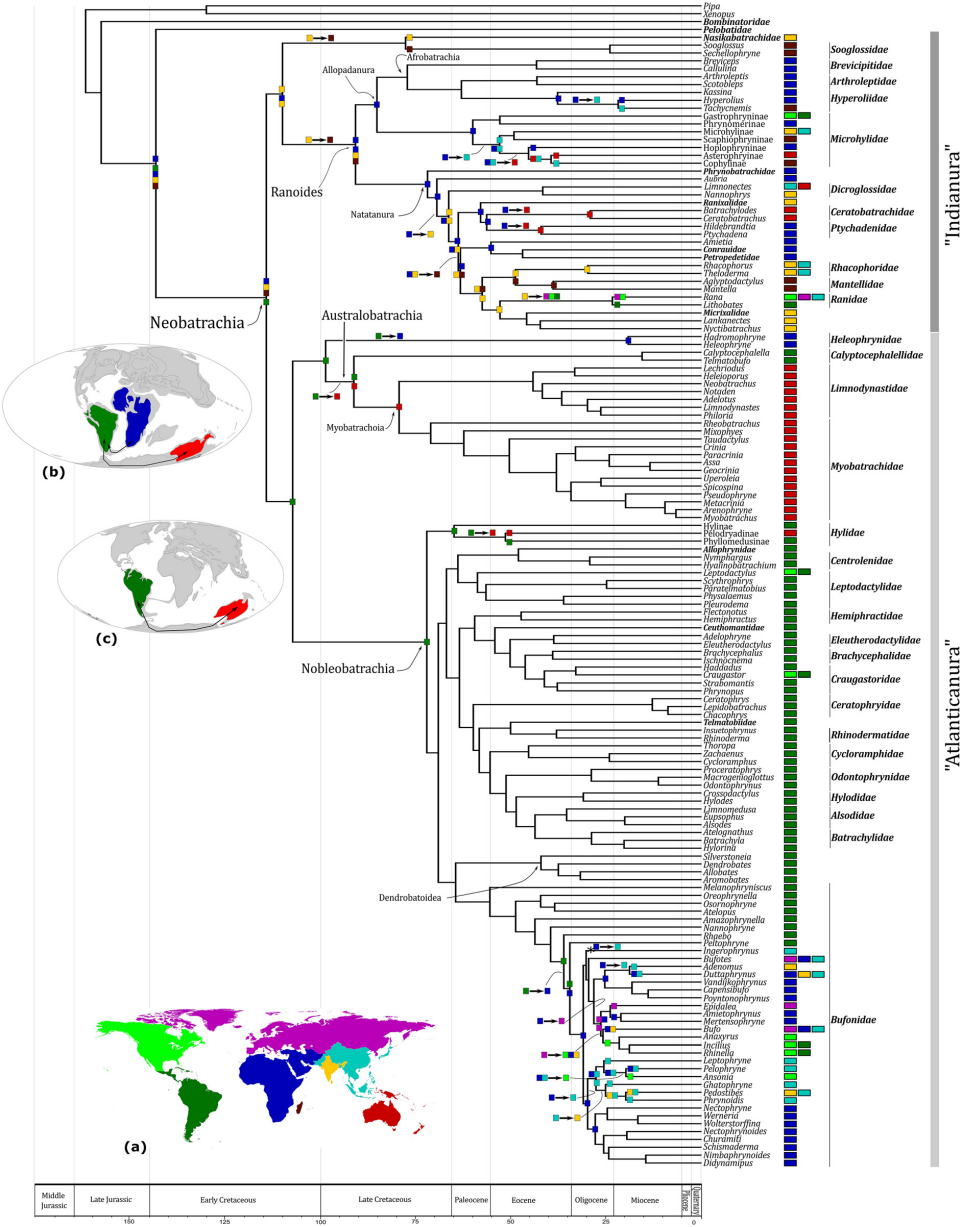
Biogeographic analysis for the diversification of Neobatrachia. Chronogram for Neobatrachia showing the biogeographic inference conducted using Lagrange. Squares on the side of tips represent the current distribution of taxa select for this study and the color is associated to the mundi map (a). Single area squares indicate an ancestor restricted to a single geographic area; combined squares indicate an ancestor with a distribution encompassing two or more areas; two squares separated by a space indicate the ancestral ranges inherited by each of the daughter lineages arising from the node. Only change of ancestral area or nodes of interest have squares and the absent means the same ancestral area of previous node. The result informed refers to more likelihood inference. Asterisk (*) represents the second bigger likelihood value. Black arrows show events and sequence of expansion of ancestral range along the branch. Late Cretaceous map (b) represents dispersal of ancestral Heleophrynidae from South America to South Africa and connection between Australia and South America by the ancestral Australobatrachia via Antarctic. Eocene map (c) represents connection between Australia and South America by the ancestral Hylidae via Antarctic.

Our time scale (Fig 1) suggests that the common ancestor of Neobatrachia and Pelobatidae lived during the Early Cretaceous (143 Ma) in Gondwanaland (Fig 2, Table 1). Our results also indicate that the diversification of crown Neobatrachia began in the mid-Cretaceous (~114 Ma; Fig 1, Table 1). In addition, it seems that all five major neobatrachian lineages arose in the Cretaceous and survived the Cretaceous-Paleogene extinction (Fig 1).

**Table 1:** Time estimates for major neobatrachian splits.

Split	Age (95% HPD)	Time scale	Ancestral range	Support
**Stem Neobatrachia**	143 (119.7–167.8)	Late Jurassic-Early Cretaceous	D|BDEF	91
**Crown Neobatrachia**	114.1 (98.2–130.8)	Early-Late Cretaceous	DEF|B	100
**Indianura**				
**Nasikabatrachidae+Sooglossidae**	109.8 (94–126.2)	Early-Late Cretaceous	E|DE	50
**Nasikabatrachidae-Sooglossidae**	77.6 (55.9–98.9)	Late Cretaceous	E|F	96
**Crown Ranoides**	90.7 (76.3–105.7)	Early-Late Cretaceous	D|DEF	100
**Allopadanura**	85.1 (70.9–99.6)	Late Cretaceous	D|D	45
**Crown Afrobatrachia**	77.1 (63.1–91.4)	Late Cretaceous	D|D	69
**Crown Microhylidae**	59.9 (46.5–74.6)	Late Cretaceous-Eocene	D|D	100
**Crown Natatanura**	71.8 (59.7–84.4)	Late Cretaceous-Paleocene	D|DEF	100
**Atlanticanura**				
**Stem Nobleobatrachia**	107.2 (92–122.7)	Early-Late Cretaceous	B|B	59
**Crown Nobleobatrachia**	72.4 (60.5–83.7)	Late Cretaceous	B|B	100
**Heleophrynidae-Australobatrachia**	98.5 (83.5–114.1)	Early-Late Cretaceous	B|B	78
**Crown Australobatrachia**	91.1 (76.7–106.1)	Early-Late Cretaceous	B|H	62
**Pelodryadinae-Phyllomedusinae**	51.4 (36.4–65.6)	Paleocene-Eocene	B|H	100
***Peltophryne* (Bufonidae)**	34.5 (28.3–40.9)	Eocene-Oligocene	B|D	36

Mean node ages (Ma) and 95% highest posterior density (HPD) obtained for Neobatrachia. The more likely ancestral range, time scale and support value (BP) are also showed. The underlined values refer to five major lineages of Neobatrachia studied here. B. Neotropic (NT) + Panamanian (PM): South America, Central America and adjacent islands; D. Afrotropical (AT) + Saharo-Arabian (S-A): all territory of African continent plus Middle East, northern Africa, except Sudan, plus Middle East; E. India and Sri Lanka (IN); F. Madagascan (MA): Madagascar and adjacent islands, including Seychelles, Mauritius and Reunion; H. Australian (AU) + Oceanian (OC): Australian region and adjacent islands, Papua New Guinea and adjacent islands.

Our phylogenetic tree suggests that Neobatrachia is divided into two major clades and the biogeographical analysis indicated a vicariant event for the crown split (Fig 2, Table 2). After the divergence, one lineage was restricted to South America and the other was confined to the African, Indian, and Madagascar regions (Fig 2). Therefore, it seems that the evolutionary history of these two major neobatrachian clades is finely coupled with events that took place in the continental crust of Gondwanaland. These events resulted in the opening of the Atlantic (105 Ma) [44] and altered the limits of the Indian Ocean.

**Table 2:** Geographical splits and divergence times for vicariant events in Neobatrachia.

Neobatrachian split	Age (95% HPD)	Geographical split (time range)
**Indianura/Atlanticanura**	114.1 (98.3–130.9)	Africa+India+Madacascar/South America (~40 Ma AF/SA [45,53])
**Indianura clade**		
**Nasikabatrachidae/Sooglossidae**	77.6 (55.9–98.9)	India/Madagascar (~90 Ma [8])
***Hyperolius*/*Tachynemis***	21.9 (11.5–31.7)	Southeast Asia/Africa
**Hoplophryninae**	45.4 (33.1–58.4)	Southeast Asia+Australia/Africa
**Asterophryinae/Cophylinae**	39.4 (27.5–57)	Southeast Asia/Australia
***Amietia*-Petropedetidae/Rhacophoridae-*Nyctibatrachus***	63.2 (52–74.3)	Africa/India+Madagascar (~170 Ma [8])
**Rhacophoridae/Mantellidae**	48.8 (38.3–60)	India/Madagascar (~90 Ma [8])
***Rana/Lithobates***	23.4 (13.2–34.6)	Holartic+Southeast Asia/South America
**Atlanticanura clade**		
**Calyptocephalellidae/Myobatrachoidea**	91.2 (76.7–106.1)	South America/Australia (~40 Ma [69,71])
**Pelodryadinae-Phyllomedusinae**	51.4 (36.4–65.7)	South America/Australia (~40 Ma [69,71])
***Epidalea*/*Amietophrynus*+*Mertensophryne***	23.9 (18.7–29.2)	Eurasia/Africa
***Bufo***	25.8 (20.7–40)	India+Africa/South America (~40 Ma AF/SA [45,53])
***Pelopryne/Ansonia***	19.8 (14.2–25.8)	Southeast Asia+Africa/South America (~40 Ma AF/SA [45,53])

Time estimates and the confidence interval for vicariant events in Neobatrachia. The time range is provided only when this information is available for a geological split. AF stands for Africa and SA for South America.

Our time estimate for this split (114 Ma, Fig 1) is very close to this geological estimate for the South American and African continental separation. Hence, we propose names for the clades that indicate this connection: Atlanticanura for the clade associated with the Atlantic Ocean events and Indianura for the clade associated with the Indian Ocean events.

### The Indianura clade

The Indianura clade includes two of the five major lineages of Neobatrachia (Fig 1), which comprise about 40% of all the diversity of living anurans [13]. Our results show a historical association between Sooglossoidea and the large Ranoides lineage [2,7,45] and indicate that this split occurred during the Early Cretaceous (~109 Ma; Fig 1, Table 1).

In our tree, the Indian family Nasikabatrachidae and Seychellois family Sooglossidae form one clade (BP 96; Fig 1), the Sooglossoidea [6]. The monophyletic status of this clade is well established [2,7,45], but our biogeographical analysis shows distinctly that the ancestor of Sooglossoidea was associated to the Indian continent (Fig 2). Then, a dispersal event would have increased the geographical distribution to the Seychelles in the Late Cretaceous (Fig 2). According to our analysis, a vicariant event resulted in the first split of the Sooglossoidea, after which the Nasikabatrachidae remained associated to the Indian continent and the Sooglossidae was confined to the Seychelles Islands (Fig 2, Tables 1 and 2).

The second major Indianura lineage was the large and cosmopolitan Ranoides (BP 100). Members of this group exhibit a remarkable morphological feature: the presence of firmly united coracoides bones [2,46]. This lineage is broadly recognized and has been recovered in many phylogenetic proposals [2,10,4]. Our results provide strong statistical support for the group.

Our results suggest that the Ranoides ancestor was probably associated to the African, Indian and Madagascar continents (Fig 2, Table 1) before the diversification of the crown Ranoides. After the main Ranoides split, the Allopadanura and the Natatanura ancestors endured on the African continent but seem to have undergone extinction in India and Madagascar during the late Cretaceous (~91 Mya).

Most allopadanuran diversity is still confined to African grounds. More recently, a few lineages in this group have dispersed to Southeast Asia (Eocene and Miocene) and probably from there to Australia (Eocene; Fig 2) where the descendants of the Asterophryinae ancestor remain to this day. Also, two sister group relationships relating African and Asian groups are likely to result from vicariant events in Allopadanura. The first is the divergence between Hoplophryninae (Africa) and the clade including Cophylinae (Madagascar) plus Asterophyinae (Australia) that took place in the middle Eocene. The second event is the split between *Hyperolius* (Africa) and *Tachycnemis* (Southeast Asia) in the early Miocene. As timing between these events fall just outside their credibility interval, it seems likely that they were prompted by distinct events (Table 2).

According to our analysis, the ancestor of the Natatanura also occupied the African region (Fig 2) after its extinction in India and in Madagascar. Different from Allopadanura, however, the descendants of the Natatanura are distributed globally. Their diversification started at the end of the Late Cretaceous (~72 Ma) and resulted in what has been known as the great Ranoides radiation (Fig 2). In the Eocene, a dispersal event took place when the ancestor of the Ceratobatrachidae spread from Africa to Australia, where the descendants remain to this day, having gone extinct elsewhere. Another dispersal event increased the geographical distribution of the Ranidae ancestor from India to the Americas and the Holartic region.

Additionally, three vicariant events are suggested by our analysis in Natatanura (Fig 2, Table 2). The first event was in the Paleocene (~63 Ma) at the split of the *Amietia*-Petropedetidae clade (Africa) from the Rhacophoridae-*Nyctibatrachus* group (India and Madagascar). In the Eocene (~50 Ma), a second vicariant event gave rise to the Rhacophoridae and Mantellidae families, which also inhabit the Indian and Madagascar region, respectively. The third vicariant event took place in the late Oligocene (~24 Ma), when the Ranidae ancestor split into the genus *Rana* (North America, Holartic and SE Asia) and the genus *Lithobates* (South America).

### The Atlanticanura clade

According to our tree, the other three major lineages of Neobatrachia are included in the newly proposed Atlanticanura clade (Fig 1), which contains the remaining almost 60% of all living anuran diversity [13]. Many members of our new clade share a unique feature in which there is no ossification of the coracoides bones. Curiously, this trait was once suggested as exclusive to the old Arcifera group, originally proposed by Boulenger in his classic monograph [46,47]. The phylogeny underlining members of the Arcifera group is available in S3 Fig.

Despite a certain similarity between members of our Atlanticanura clade and those of Boulenger’s Arcifera group, many critical differences are evident [46] (Fig 1). For instance, this feature is found not only among neobatrachians, a currently well-established clade, but also in some archeobatrachian lineages such as discoglossideans and pelobatideans [46]. On the other hand, it is absent in Atlanticanuran (e.g., the Australobatrachia and in some nobleobatrachians) and in Indianura lineages (e.g., Ranoides), suggesting a multiple origin for this trait.

In some studies, the Heleophrynidae family has been recovered as the sister group of the remaining neobatrachians [2,5,10,41,43], but that was not the case here (Fig 1) (see also [7,45] for alternative hypotheses). According to our time-tree, the South American ancestor of the Atlanticanura clade split into the African family Heleophrynidae (BP 100) and the Australobatrachia (BP 62) in the Early Cretaceous (~107 Ma; Figs 1 and 2). In this case, the South American Heleophrynidae ancestor probably augmented its geographical distribution to the African continent before the diversification of the group in Africa (Fig 2). On the other hand, the Australobatrachia ancestor expanded the distribution to Australia before splitting into the Chilean Calyptocephalellidae family (BP 100) and the Australian Myobatrachoidea clade (BP 100).

The diversification of the Australobatrachia lineage started about 91 Ma and the ancestor expanded its geographical distribution to the Australian continent before the first split of Australobatrachian anurans (Fig 2, Table 1). According to our biogeographical results, the split between the Chilean Calyptocephalellidae family and the Australian Myobatrachoidea lineage occurred due to a vicariant event (Fig 2, Table 2). Our results suggest that the Calyptocephalellidae remained associated to the South American continent, whereas the Myobatrachoidea was connected to the Australian continent. The event might have required an austral connection via the Antarctic continent.

The diversification of crown Nobleobatrachia began in the Late Cretaceous (~72 Ma). This group includes the second great Atlanticanura lineage and probably originated on the South American continent, as our biogeographical analysis suggests (Fig 2). Indeed, the vast majority of nobleobatrachians is currently associated to the South and Central American continents. One example of a vicariant event in this lineage was associated with the Pelodryadinae and Phyllomedusinae subfamilies of Hylidae (Fig 2, Tables 1 and 2). Just like all other nobleobatrachians, the Hylidae ancestor was restricted to the South American continent. However, after the divergence of Hylinae, a dispersal event possibly took place when the ancestor of Pelodryadinae and Phyllomedusinae expanded its distribution to Australia. As the ancestral area of this ancestor included South America and Australia, it is most likely that after the split, Pelodryadinae became restricted to Australia, whereas Phyllomedusinae was confined to the Neotropical region (Fig 2).

The other conspicuous biogeographical event was inferred in the bufonids, a series of dispersal and extinction events that are thought to explain the diversification of the family (Fig 2). In that case, a paraphyletic series of South American endemic lineages is interrupted by a monophyletic group that inhabits the Old World, mainly Africa. According to our analysis, before the divergence of the last South American lineage that leads to *Peltophryne* (~35 Ma), the ancestor of this bufonid group expanded its geographical distribution to the African continent (Fig 2, Table 1). The *Peltophryne* ancestor persisted in the Neotropical region, whilst the sister group remained on the African continent after the vicariant event. Ever since, a complex series of events of biogeographical connection and disconnection occurred between areas that include colonization of Southeast Asia, India and the Holartic (see Fig 2, Tables 1 and 2).

## Discussion

Our time-tree estimates agree with geological events that have been reported for the geographical areas of the neobatrachian lineages (Fig 1, Tables 1 and 2). For instance, our estimate for the vicariant event on the diversification of crown Neobatrachia (~114 Ma) into Indianura and Atlanticanura is highly congruent with the primary rupture between the South American and African continents in the Cretaceous. In this case, sea floor spreading started in the Early Cretaceous (135 Ma) but the final physical separation between South Africa and South America took place around ~105 Ma [44]. Hence, our time estimate for the split supports the hypothesis that the Atlanticanura ancestor remained in the South American region, whereas the Indianura ancestor was confined to the African lands east of the Atlantic Ocean after the physical separation between these continents.

The association of the early neobatrachian diversification and the opening of the Atlantic Ocean has been recently reported in a comprehensive biogeographical study for amphibians [11]. Our main topological results, however, remain unparalleled since another major neobatrachian split was also explained by the Indianura vicariant event. This emphasizes the importance of vicariant events in the early neobatrachian diversification. Additionally, Pyron’s time estimate for the main neobatrachian split (152 Ma) [11] falls outside our own estimate range (98–131 Ma; Fig 1). As our time estimate agrees well with the geological information for Atlantic Ocean opening [44], the time difference between these split might be explained due to different calibration schemes.

A second event relating these great landmasses would be the dispersal of the Heleophrynidae ancestor from South America to South Africa, which might have occurred in the Cretaceous, according to our time scale (~98 Ma; Table 1). Heleophrynidae is currently represented by seven described species that are restricted to the southernmost region of South Africa [13]. Our estimated age for the divergence of Heleophrynidae is very close to the final disconnection between southern South America and southern Africa during the Early Cretaceous [44,48].

Curiously, the phylogenetic position for the Heleophrynidae varies widely among the current neobatrachian hypotheses [2,5,7,10,41,43,45]. Such variation, may possibly be related with long branch attraction artifacts [49], perhaps due to massive extinction events in this lineage. In our proposition, however, the strong biogeographical association represents an interesting twist on the study of this relictual South African family despite the relatively low support (Fig 1). More comprehensive studies using a larger taxon sampling and genomic sequence data might be able to estimate a more accurate divergence scenario for the Heleophrynidae.

In our time-scale, we find a third geographical split involving African and South American landmasses in microhylids (Fig 2). In this case, however, the time estimate between Gastrophryninae (America) and Phrynomerinae (Africa) subfamilies is much more recent (Eocene ~52 Ma). Similarly, recent estimates were also found between African and South American groups of primates, lizards and birds [50,51,52]. A geological study indicated that gene flow between populations in these landmasses might have been possible through an enduring chain of islands that would have connected Africa and South America until more recently [53]. That study, based on magnetic anomalies, indicated that this chain of islands started to break apart around 40 Ma, only then interrupting the passage from Africa to South America [53].

Nonetheless, why the chain of islands allowed gene flow between some groups but nor others remains to be explained. On the one hand, we have a Cretaceous estimate for the Indianura and Atlanticanura split (and the dispersal of the Heleophrynidae ancestor) that is congruent with the divergence of African and South American clades of Allosauroidea dinosaurs [54]. On the other hand, we have microhylid frogs that, along with primates, passerine birds and lizards, managed to maintain gene flow between inhabitants of these landmasses until the Paleogene.

It is possible that this difference in timing is related to the distinct ecological constraints of the ancestors of these two groups. For instance, large-bodied carnivorous dinosaurs [55] such as the Allosauroidea might not have been able to survive on the chain of islands, due to the limited availability of prey. Earlier anurans probably also showed restricted survival capabilities, likely because they were highly dependent on surface water for reproduction, which was probably a limited resource in the chain of islands [56]. These restrictions, therefore, would have prevented the use of the island chain to maintain gene flow for dinosaurs and for early neobatrachians.

Conversely, life in the chain would be more viable for members of the second group. For instance, birds might have been able to fly over small portions of seawater, increasing the probability of survival in that environment. Also, small amniotes such as lizards and primates, might have been able to find sufficient food, and surface water would not be as critical due to the amniote egg.

Microhylids do not have amniote eggs, but their ancestors still managed to maintain gene flow across the separating continents. Some alternative reproductive strategies have been reported in microhylids, such as direct development with no tadpole stages. This strategy was recorded in the Gastrophryninae subfamily, which is directly related to the node in question [57,58]. Therefore, if ancestors of this clade shared this trait, it would have made them more prone to survival in the Atlantic island chain (see [58] for a more ancient divergence time for microhylids).

One alternative explanation may not be ruled out, as the Gastrophryninae subfamily is not restricted to South America and also inhabits the North American continent. Therefore, it is possible that northern alternative land bridges would also explain this pattern. One possibility is the North Atlantic land bridge that enabled the crossing of mammals from Europe to North America though the Eocene [59]. Climate issues, however, might have prevented the use of this northern passage for frogs.

In the Indianura clade, the divergence times estimated here are also congruent with geological events. The original authors of Nasikabatrachidae suggested a split date for the Indian and Madagascan lineages of Sooglossoidea in the Early Cretaceous (131 Ma) [7] associating it with the break between these landmasses. Our estimated divergence time between the Nasikabatrachidae and Sooglossidae families, however, indicated a much more recent date for this split in the Late Cretaceous (77 Ma, Fig 1) (see also [11] at 99 Ma and [4] at 101 Ma for earlier estimates for this split).

A recent geological study suggested a different scenario for the isolation of the Indian plate based on biostratigraphic and geological data [8]. In their study, the authors suggested that during most of the Cretaceous, India and the Seychelles Islands still maintained a land connection through the Laxmi bridge. The first break between these lands occurred around 70 Ma, when the Seychelles and the Laxmi bridge were first isolated from India. Five million years later, the Laxmi bridge disconnected from the Seychelles, drifted towards India, and joined the Indian plate around 65 Ma, finally separating these landmasses. Therefore, our results for the Indianuran Nasikabatrachidae and Sooglossidae split date this divergence to the late Cretaceous (77 Ma), which is more consistent with more recent geological estimates for the geological events separating the Seychelles and India.

The other major Indianura lineage is the Ranoides. Currently, two distinct hypotheses have been proposed to explain the origin and the diversification of the Ranoides. In the first (the out-of-Africa hypothesis), the Ranoides ancestor appeared in Africa and then reached other continents [60,61]. This proposal is supported by biostratigraphical data, as the oldest record of Ranoides dates from Cretaceous sediments in Africa [57,61,62].

The alternative hypothesis is the out-of-India hypothesis [60,63]. According to this hypothesis, the ancestor of this clade would have dispersed via India when the Indian plate separated from the main Gondwana and drifted towards continental Asia during the Early Cretaceous. When the plate reached Southeast Asia, around 50 Ma, the Ranoides lineage spread to other regions in the Paleogene. Our biogeographical analysis does not resolve this debate, as India and Africa are part of the ancestral area of the Ranoides (Fig 2).

The out-of-India hypothesis, however, was originally proposed based on a much earlier divergence time for the Ranoides split [60,63]. These authors were motivated by the complex geological history of Indian and the Seychelles landmasses, which was undisclosed at the time. In fact, in order to date the neobatrachian split, those authors calibrated their tree assuming that the (Madagascar) Mantellidae and (Indian) Rhacophoridae families diverged around 87 Ma [60]. As previously stated, the use of biogeographical events as calibrations may introduce a bias to the time-tree analysis [64].

Also, our results indicate that the core Ranoides split (between Allopadanura and Natatanura) (~90 Ma) took place when India was still in the middle of the Indian Ocean, well isolated from other landmasses in the Cretaceous. Hence, these results would point to an African origin for Ranoides. Furthermore, our biogeographical results better fit the interpretation that Ranoides originated in Africa, with its ancestor widely distributed in Africa, India, and Madagascar around 90 Ma, assuming the ancestor in India and Madagascar became extinct before the diversification of Allopadanura and Natatanura (see Fig 2). Given the out-of-Africa scenario, at the time of the early diversification of Ranoides, some lineages remained endemic in Africa, while others would have spread to other continents (see also [11]).

In fact, our results also show that the split between Rhacophoridae (India and SE Asia) with Mantellidae (Madagascar) (~48 Ma) occurred in the Eocene. This timeframe is somewhat intriguing, as India was probably isolated from Madagascar since 65 Ma and from other lands since the Cretaceous. According to a recent hypothesis, India remained an isolated plate until 50 Ma, when northward rifting subducted the plate under Asia [8].

Finally, our biogeographical analysis indicates two vicariant events involving the South American and Australian regions: the first took place when Calyptocephalellidae (Chile) and Myobatrachoidea (Australia) split, and the second during the split between Phyllomedusinae (South America) and Pelodryadinae (Australia). Timing for these divergences was quite different: the first took place in the Late Cretaceous, whereas the second occurred in the Eocene, suggesting these were independent events. Drastic climatic changes have been described during these periods [65,66], including sea levels reaching the highest marks of the last 100 Ma [67,68]. It seems likely that successive events of connections and disconnections between South America and Australia might have happened during those time periods, driving new waves of speciation in neobatrachians. The reticulate history of South American and Australian endemic groups was probably maintained via the Antarctic continent but would have ended at the Eocene, when a shallow seaway between Australia and Antarctica might have influenced faunal interchanges [69,70,71]. Other taxonomic groups present the same biogeographic pattern, supporting the notion of connections between these continents, as presented in our study. These groups include invertebrates [69,72], plants [73,74], and other vertebrates [71,75].

In the Nobleobatrachia lineage, the quick diversification followed by a geographical expansion inferred for Bufonidae is noteworthy [11,76]. Curiously, in spite of the great worldwide distribution of Bufonids, no lineages are endemic to Australia, Madagascar or other oceanic regions. This is probably because these areas had already disconnected completely when the ancestor expanded the range, which was highly dependent on the availability of eggs and tadpoles (see recontruction of reproductive mode by [56]).

This high speciation rate is associated with the Oligocene period (see [76] for earlier estimate), a time when the Earth was undergoing the first Cenozoic glaciation [66,77,78,79]. Interestingly, morphological features associated with reproduction seem to reflect adaptation that allowed the colonization of new environments by Bufonidae, which would have been concomitant with the expansion of Bufonidae to the Old World [80]. As the presence of water is critical for anuran maintenance, the rapid diversification must have been associated with the constant effect of natural selection due to expansion of ancestral distribution during dry and cold periods [80,81].

In this paper, we investigated the biogeographical events that influenced the evolution of the most diverse lineage of living anurans. Our time scale indicates that the diversification of Neobatrachia was tightly associated with the Gondwana supercontinent during the Cretaceous period. Also, we show that climatic and geological changes contributed to the remarkable biogeographical events that determine the current distribution of major neobratrachian lineages. Further details, in particular of the minor scale, are still unclear, and must be clarified in order to pose a more detailed scenario for Neobatrachia evolution.

## Supporting Information

S1 Fig Unrooted tree for Neobatrachia with no outgroups. Maximum likelihood tree resulted from 12 makers analysis excluding the outgroups. Numbers above nodes represent the bootstrap support values. Red branches are Indianura lineages whereas blue branches are included in the Atlanticanura clade.
(PDF)

S2 Fig Maximum likelihood tree of Neobatrachia and fossil calibration. Maximum likelihood tree resulted from 12 makers here used. The numbers on the node represent the bootstrap support values. The names at the node show the major lineages here discussed. Red circles show the calibration points and the numbers at these represent each calibration that was described above.
(TIF)

S3 Fig Representation of Arcifera group. Chronogram for Neobatrachia derived from Bayesian analysis employing a relaxed molecular clock using BEAST and fixing the topology reconstructed by Maximum Likelihood analysis. Stars next nodes indicate the bootstrap value ≥ 95. Bootstrap values lower than 95 are shown next to corresponding nodes. The horizontal blue bars represents 95% of highest posterior density (HPD). The highlight branches (bold branches) represent the five major lineages of Neobatrachia. Red branches represent Arcifera group whereas green branches represent Firmisternia group according Boulenger (1982). The time scale below chronogram measures time in millions of years.
(TIF)

S1 File Data Partitions. PartitionFinder result.
(TXT)

S2 File Biogeographic analysis information. Information about fossil calibrations, biogeographical areas, probability of distribution (Q matrix), and time slices (TS).
(DOC)

S1 Table Genbank accession numbers. GenBank accession numbers for each species analysed.
(DOC)

S2 Table Geographical distribution and taxonomic information. Geographical distribution and taxonomic information for each species analysed.
(DOC)
