# Supplementary figures and images for: The Gondwana Breakup and the History of the Atlantic and Indian Oceans Unveils Two New Clades for Early Neobatrachian Diversification
Source: PLoS One. 2015 Nov 30;10(11):e0143926. doi: 10.1371/journal.pone.0143926 (PMC4664409; doi:10.1371/journal.pone.0143926)

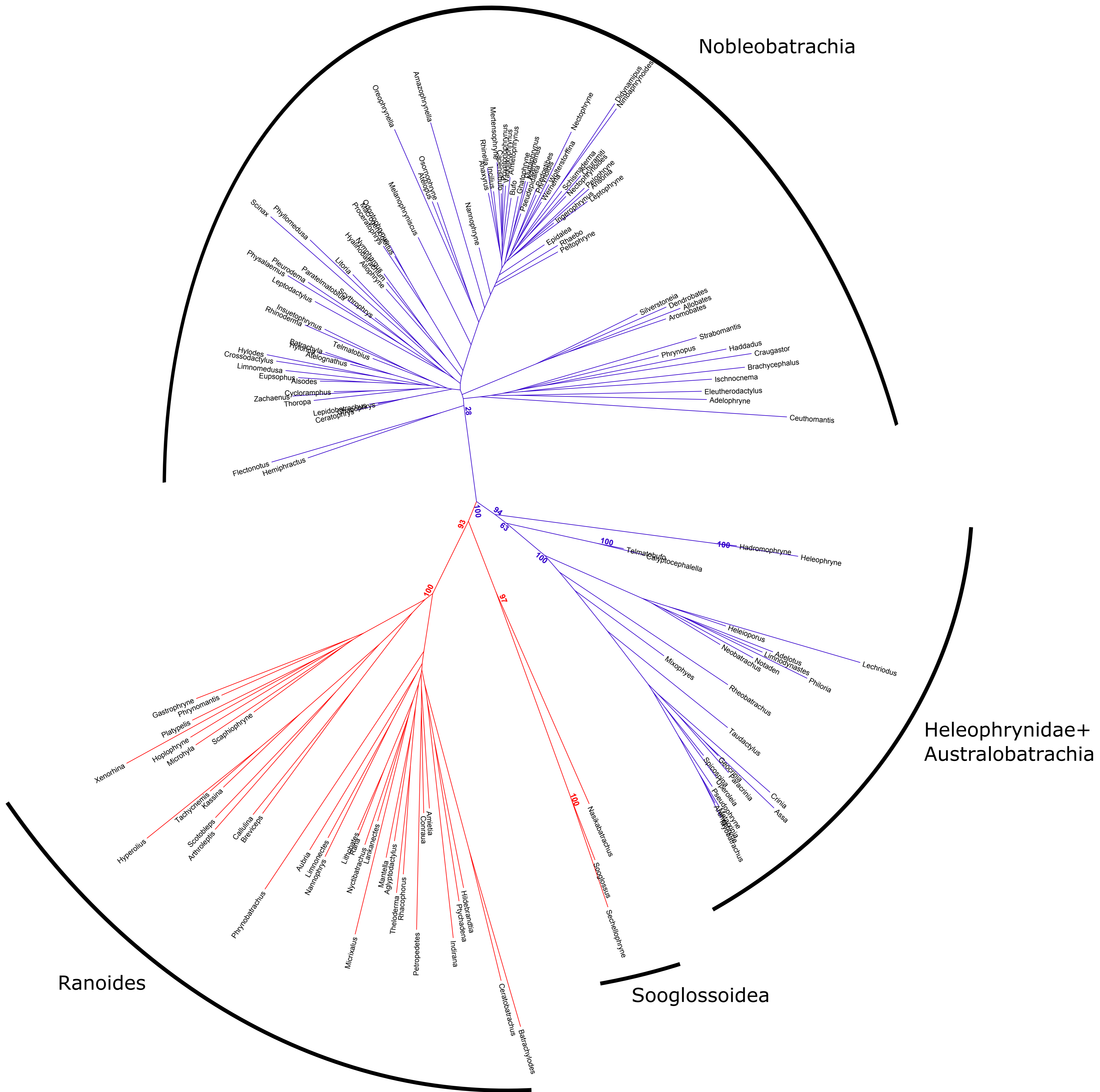

Supplement: S1 Fig — Maximum likelihood tree resulted from 12 makers analysis excluding the outgroups. Numbers above nodes represent the bootstrap support values. Red branches are Indianura lineages whereas blue branches are included in the Atlanticanura clade. (PDF) [file pone.0143926.s001.pdf]

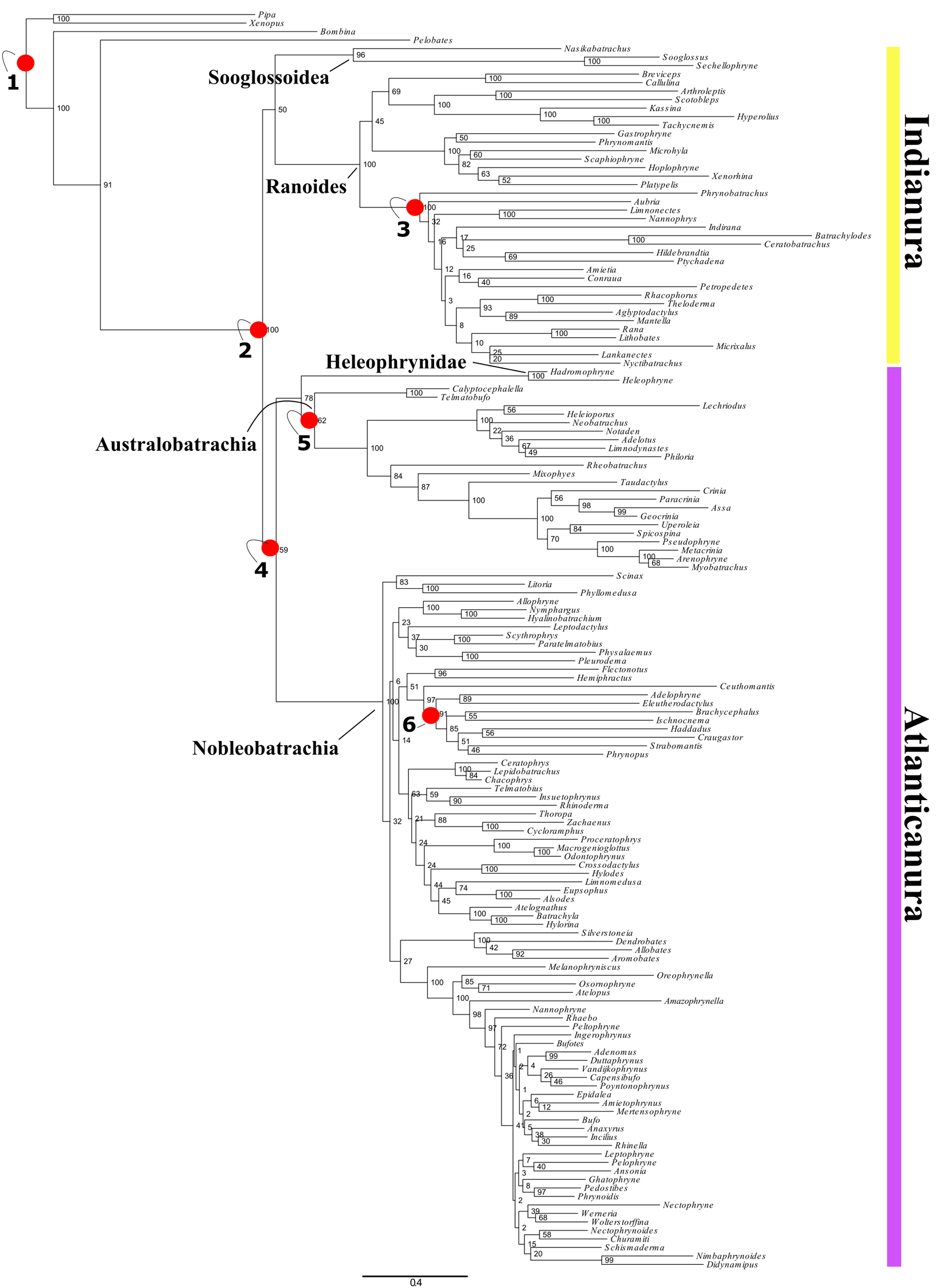

Supplement: S2 Fig — Maximum likelihood tree resulted from 12 makers here used. The numbers on the node represent the bootstrap support values. The names at the node show the major lineages here discussed. Red circles show the calibration points and the numbers at these represent each calibration that was described above. (TIF) [file pone.0143926.s002.tif]

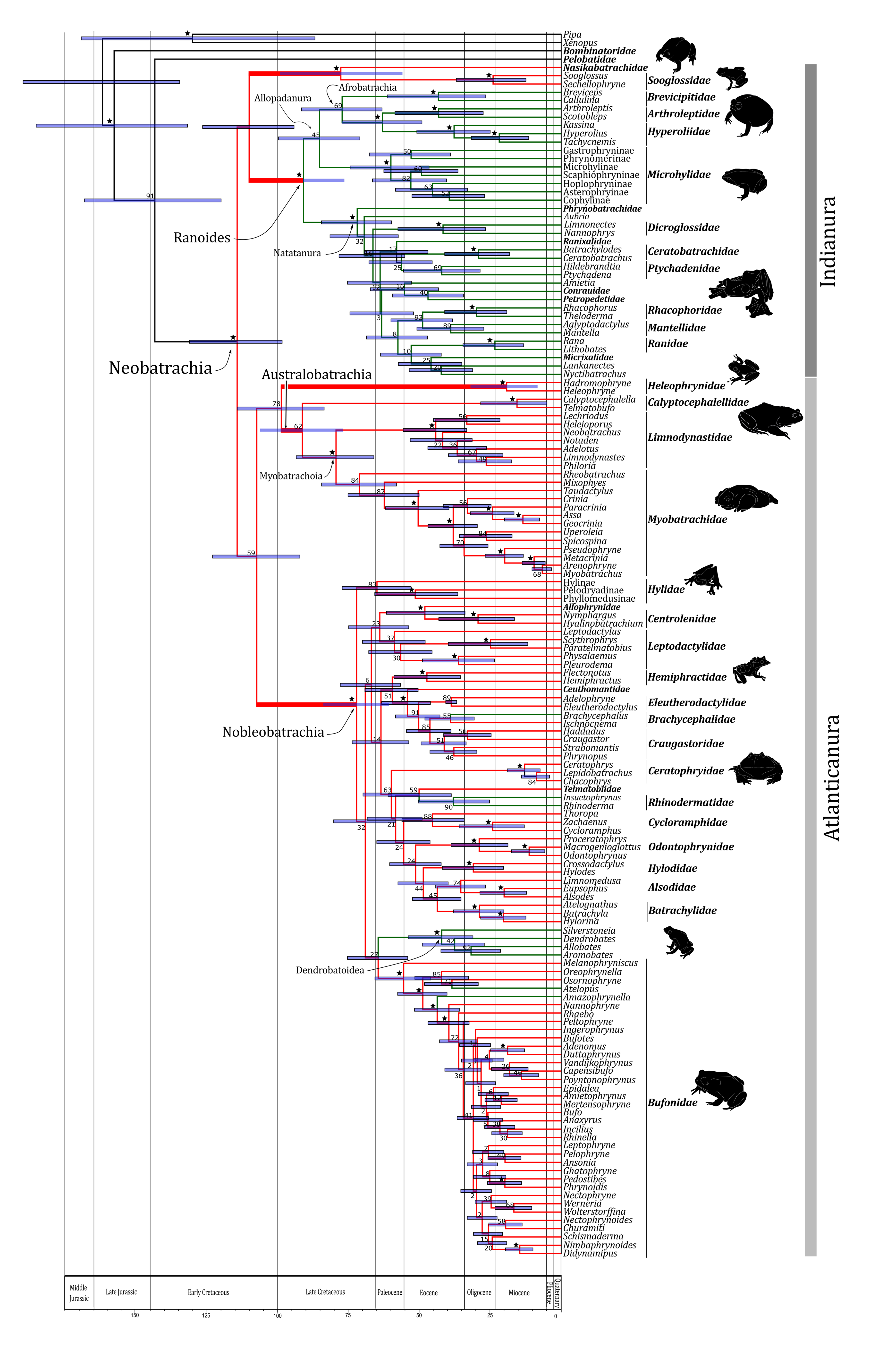

Supplement: S3 Fig — Chronogram for Neobatrachia derived from Bayesian analysis employing a relaxed molecular clock using BEAST and fixing the topology reconstructed by Maximum Likelihood analysis. Stars next nodes indicate the bootstrap value ≥ 95. Bootstrap values lower than 95 are shown next to corresponding nodes. The horizontal blue bars represents 95% of highest posterior density (HPD). The highlight branches (bold branches) represent the five major lineages of Neobatrachia. Red branches represent Arcifera group whereas green branches represent Firmisternia group according Boulenger (1982). The time scale below chronogram measures time in millions of years. (TIF) [file pone.0143926.s003.tif]
