# Supplementary material for: The Gondwana Breakup and the History of the Atlantic and Indian Oceans Unveils Two New Clades for Early Neobatrachian Diversification
Source: PLoS One. 2015 Nov 30;10(11):e0143926. doi: 10.1371/journal.pone.0143926 (PMC4664409; doi:10.1371/journal.pone.0143926)
Supplement: S2 File — Information about fossil calibrations, biogeographical areas, probability of distribution (Q matrix), and time slices (TS). (DOC) [file pone.0143926.s005.doc]

**Supporting Information 2:** Information about fossil calibrations, biogeographical areas, probability of distribution (Q matrix), and time slices (TS).

1. **Fossil calibrations**

The maximum likelihood tree and the position of calibrations are showed in Fig 1. The calibration points and distribution settings were used as priors are described bellow:

1. **Xenoanura-Sokolanura split (root calibration):** normal distribution ranging from 145 Ma as minimum age for *Rhadinosteus parvus* [1] and 251 Ma for maximum age for *Triadobatrachus* *massinoti* [2]; mean=198,25; stdev=32,1.
2. **Neobatrachia crown group:** normal distribution ranging from 99,6 Ma as minimum age for *Cratia gracilis* [3] and 161,2 Ma as maximum age for *Rhadinosteus parvus*; mean=130,4; stdev=18,7.
3. **Natatanura crown group:** normal distribution ranging from 34 Ma as minimum age for *Thaumastosaurus gezei* [4] and 125 Ma as maximum age for *Arariphrynus placidoi* [5]; mean=79,5; stdev=27,5.
4. **Nobleobatrachia-Sister Group split:** normal distribution ranging from 83,5 Ma as minimum age for *Baurubatrachus pricei* [6] and 125 Ma as maximum age for *Eurycaphalella alcinae* [3]; mean=104,25; stdev=12,6.
5. **Myobatrachidae-Limnodynastidae split:** normal distribution ranging from 65,5 Ma as minimum age for *Indobatrachus pisillus* [7] and 125 Ma as maximum age for *Cratia gracilis* [3]; mean=95,25; stdev=18.
6. **Eleutherodactylidae-Sister Group split:** normal distribution ranging from 34 Ma as minimum age and 40 Ma as maximum age for *Eleutherodactylis* sp. [8]; mean=38,5; stdev=1,0.
7. **Biogeographical Analysis - Lagrange**

***Biogeographical areas***

The biogeography areas used here were adapted from [9] (Fig 1) for present distribution of Neobatrachia. We performed some adaptations of Holt’s areas: (i) the “India” area was separated from “Oriental” area because the endemism of some Neobatrachia lineages and (ii) the areas here designated as “B”, “D”, “G”, and “H” represent joined areas. Biogeographical areas used here for analysis using Lagrange are described below.


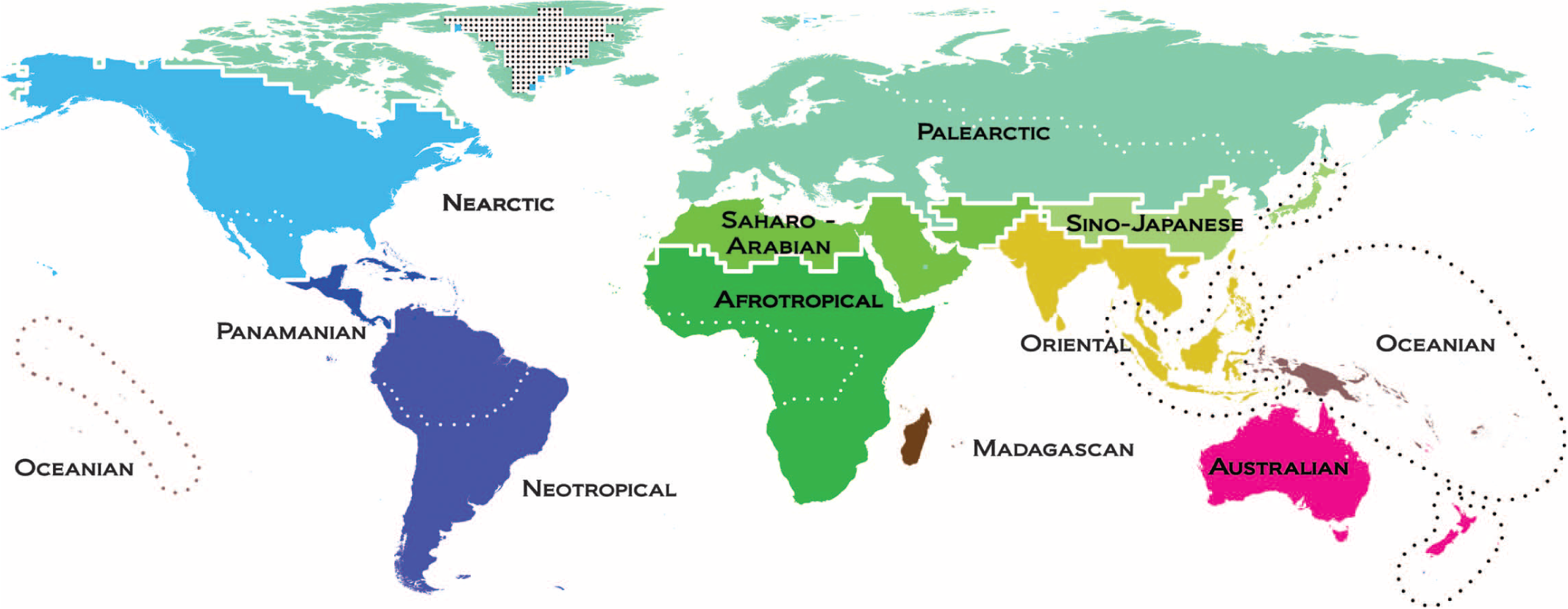


**Fig 1:** Biogeography areas defined by Holt *et al.* [9].

1. Nearctic (NA): North America, except South Mexico and northern end of Canada and Greenland;
2. Neotropic (NT) + Panamanian (PM): South America, Central America and adjacent islands;
3. Palearctic (PA): all European and Asian countries, except Southern of China, Southeast Asia, India, Bangladesh, Bhutan, Japan, Nepal, Pakistan and Sri Lanka;
4. Afrotropical (AT) + Saharo-Arabian (S-A): all territory of African continent plus Middle East, northern Africa, except Sudan, plus Middle East;
5. India and Sri Lanka (IN);
6. Madagascan (MA): Madagascar and adjacent islands, including Seychelles, Mauritius and Reunion;
7. Oriental (OR) + Sino-Japanese (S-J), excluding India and Sri Lanka: Southeast Asia plus Bangladesh, Bhutan, Nepal and Pakistan, Tibetan region, southern China and Japan;
8. Australian (AU) + Oceanian (OC): Australian region and adjacent islands, Papua New Guinea and adjacent islands.

**Time Slices and Q matrixes**

The time slices (TS) are following. Each TS have duration of 30 Ma.
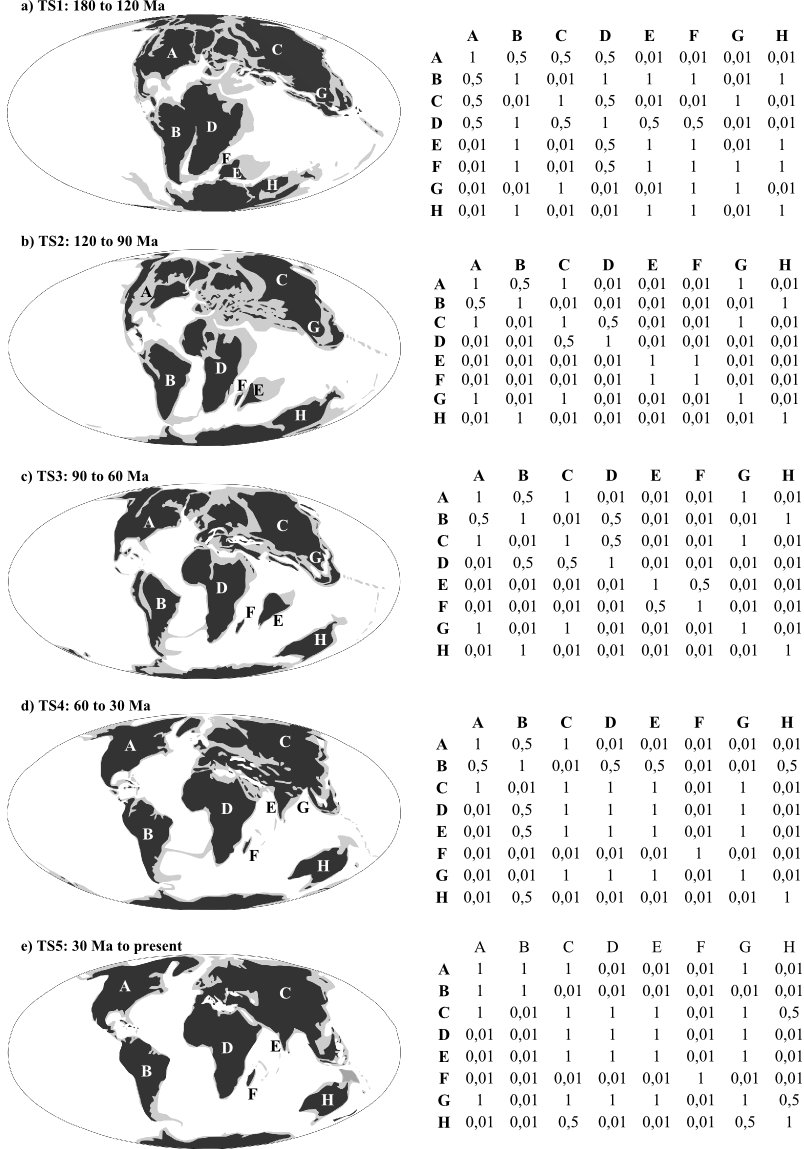


Fig 2: Palaeogeographical model used in Neobatrachia biogeographical analysis using Lagrange. The five time slices (TS) are shown with respective Q matrixes, providing the connectivity between the biogeographical areas along the time.

#### TS1: between 180 and 120 Ma (Middle-Late Jurassic to Barremian, Early Cretaceous, Fig 2a).

In this first TS, the Pangaea break up to Gondwana and Laurasia have already started since 180 Ma. The open of Central Atlantic Ocean disconnected the Western Gondwana and Laurasia, what did not allow any mainland route [10-12]. The mainland composed by Palearctic and Nearctic, which comprised the northern continent, the Laurasia, remained joined by connection between Europe and Greenland via British Isles in the middle of a narrow Atlantic Ocean [12]. The southern continent, the Gondwana, was composed by South America, Africa, Antarctic, Australia, India, Seychelles and Madagascar, which remained connected up to approximately 170 Ma, when the eastern Gondwana (Antarctica + Australia + India + Seychelles + Madagascar) began the break up from western Gondwana (South America + Africa). The second Gondwana break up was nearly 167 Ma, when Africa separated from India-Madagascar-Antarctica. Following, India-Madagascar broke up from Antarctic-Australia around 130 Ma [13]. South America and Africa stayed connected [14,15] up to Eocene [16]. This connection allowed a possible dispersal route from Africa to Australia, via South America. It is possible that dispersal between Laurasia and Gondwana began decrease in this period occurring only by islands [12].

#### TS2: between 120 and 90 Ma (Aptian, Early Cretaceous to Turonian, Late Cretaceous, Fig 2b).

In that TS, Laurasia and Gondwana had already separated. There was a continental shelf linking these two areas. However, the sea level increased in that period what which made it more difficult for connection even islands in this region [12]. The northern Atlantic Ocean began rise at Late Cretaceous nearly 90 Ma. Before the opening that ocean, the dispersal between Nearctic and Palearctic happened by land bridges, the Bering land bridges, since around 100 Ma up to Pliocene (~3,5 Ma; the connection by Bering land bridge existed in cycle along the Pleistocene too), and by North Atlantic land bridge (NALB) since the transition Cretaceous Paleogene. These connections via NALB remained up to, at least, Miocene [11,17,18-20]. These connections, as by Beringian bridge as NALB, allowed dispersal between Palearctic and Nearctic during all TS since Aptian, at Early Cretaceous (TS2-TS5). (Fig 2b-e). During TS2 the opening of South Atlantic began the rupture between Africa and South America, which were disconnected since around 100 Ma [21]. Nevertheless, there are geological evidences that suggest a connection between Africa and South America via islands up to Eocene, nearly 40 Ma [16,22-25] (Fig 2b-d). There were connection between Neotropic and Nearctic areas via different ways by islands, for instance the geological evidence of existence of Gaarland at Eocene-Oligocene transition (~35 to 33 Ma) [10], since TS2 up to TS4. During TS2 also happened the disjoin between India and Madagascar nearly 90 Ma being Seychelles still linked to India (Fig 2b) [13].

#### TS3: between 90 and 60 Ma (Turonian, Late Cretaceous to Danian, Paleocene, Fig 2c).

It is possible that in that TS existed the rupture between Africa and Palearctic by high level of sea at end of Cretaceous. However, the subsequent dispersal among Africa and Palearctic was possible by connections via islands during periods with low levels of sea. There are evidences of dispersal possibility with fossil groups up to Eocene (~36 Ma) [22]. The disconnection between India and Seychelles happened at TS3. Seychelles was joined to India by Laxmi ridge. Nearly 70 Ma Laxmi ridge with Seychelles broke up of India. In a second time, the Laxmi ridge disjoined from Seychelles and reconnected with India. In that time, India plus Laxmi ridge disconnected definitively from Seychelles, around 65 Ma [13].

#### TS4: between 60 and 30 Ma (Danian, Paleocene to Rupelian, Early Oligocene, Fig 2Error: Reference source not foundd).

During the TS4, the African continent connected to Palearctic due close of Tethys seaway. In that period, India moved rapidly in direction of southern Palearctic where collided with Southern Asian nearly 52 Ma. As previously described, the connection between South America (B) and Australia (H) via Antarctic remained since Pangea up to 40 Ma. This more recent connection was possible due the sea transgressions and via Tazmania [15]. At the Late Eocene all continental landmass were disjoined (Fig 2d).

#### TS5: between 30 Ma and the present (Rupelian, Early Oligocene to Neogene, Fig 2e).

During the last TS here defined, the Earth was on the expressive climatic chances, what influenced the global temperature and sea level [12]. In this panorama, areas that before did not connected began to be. Australia (H) and Oriental (G) regions began to connected by Pacific Islands [26-28] and possible dispersal should have happened, despite Himalaya uplift around 10 Ma [13]. During this TS, the connection between Neotropic and Nearctic areas also happened. The Caribbean Islands formed and Panamanian Isthmus was formed up to Pliocene [10,29]. Other possible dispersal route that emerged were the volcanic islands existent along the Mozambique channel joining Madagascar Island and Africa since Langhian, Late Miocene around 15 Ma [30,31].

After the definition of dispersal matrixes, the script was generated and it was run using Lagrange program in local computer.

**References**:

1. Henrici AC. A New Pipoid Anuran from the Late Jurassic Morrison Formation at Dinosaur National Monument , Utah. J Vertebr Paleontol. 1998; 18(2): 321–332.

2. Rage J, Rocek Z. Redescription of Triadobatrachus massinoti (Piveteau, 1936) an anuran amphibian from the early Triassic. Palaeontographica Abt A. 1989; 206: 1–16.

3. Báez AM, Moura GJB, Gómez RO. Anurans from the Lower Cretaceous Crato Formation of northeastern Brazil: implications for the early divergence of neobatrachians. Cretaceous Res. 2009; 30(4): 829–46.

4. Laloy F, Rage J-C, Evans SE, Boistel R, Lenoir N, et al. A re-interpretation of the Eocene anuran Thaumastosaurus based on microCT examination of a “Mummified” specimen. PloS One. 2013; 8(9): e74874.

5. Leal MEC, Brito PM. Anura do Cretáceo Inferior da Bacia do Araripe, Nordeste do Brasil. In: Gallo V, Brito PM, Silva HMA, Figueiredo FJ. Paleontología de Vertebrados: Grandes Temas e Contribuções Científicas. Rio de Janeiro: Interciencia; 2006. pp. 145–152.

6. Báez AM, Perí S. *Baurubatrachus pricei*, nov. gen. et sp., un anuro del Cretácico Superior de Minas Gerais, Brasil. An Acad Bras Ciênc. 1989; 61: 447–458)

7. Noble G. The fossil frogs of the intertrappean beds of Bombay, India. Am Mus Novit 1930; 401: 387–389.

8. Poinar GO, Cannatella DC. An upper eocene frog from the dominican republic and its implication for Caribbean biogeography. Science. 1987; 237(4819): 1215–1216.

9. Holt BG, Lessard J-P, Borregaard MK, Fritz SA, Araújo MB, et al. An Update of Wallace’s Zoogeographic Regions of the World. Science. 2013; 339: 74–77.

10. Iturralde-Vinent MA, MacPhee RDE. Paleogeography of the Caribbean region: Implications for Cenozoic biogeography. B Am Mus Nat Hist. 1999; 238: 1-95.

11. Sanmartín I. Patterns of animal dispersal, vicariance and diversification in the Holarctic. Biol J Linn Soc. 2001; 73(4): 345–390.

12. Scotese CR. PALEOMAP website. 2002. Available: http://www.scotese.com

13. Chatterjee S, Goswami A, Scotese CR. The longest voyage: Tectonic, magmatic, and paleoclimatic evolution of the Indian plate during its northward flight from Gondwana to Asia. Gondwana Res2013; 23(1): 238–67.

14. Almeida EAB, Pie MR, Brady SG, Danforth BN. Biogeography and diversification of colletid bees (Hymenoptera: Colletidae): emerging patterns from the southern end of the world. J Biogeogr. 2012; 39(3): 526-44.

15. Woodburne MO, Case JA. Dispersal, vicariance, and the Late Cretaceous to early tertiary land mammal biogeography from South America to Australia. J Mamm Evol 1996; 3(2): 121–61.

16. Oliveira FB, Molina EC, Marroig G. Paleogeography of the South Atlantic: A route for primates and rodents into the New World? In: Garber PA, Estrada A, Bicca-Marques JC, Heymann EW, Strier, KB. South American primates, developments in primatology: Progress and prospects. New York: Springer New York; 2009. pp. 55–68.

17. Denk T, Grímsson F, Zetter R. Episodic migration of oaks to Iceland: Evidence for a North Atlantic “land bridge” in the latest Miocene. Am J Bot. 2010; 97(2): 276–87.

18. Denk T, Grimsson F, Zetter R, Símonarson LA. The Biogeographic History of Iceland – The North Atlantic Land Bridge Revisited. In Late Cainozoic Floras of Iceland. Dordrecht: Springer Science+Business Media BV; 2011.pp. 647-68.

19. Tiffney B. Perspectives on the origin of the floristic similarity between eastern Asia and eastern North America. J Arnold Arbor. 1985a; 66: 73–94.

20. Tiffney B. The Eocene North Atlantic land bridge: Its importance in Tertiary and modern phytogeography of the Northern Hemisphere. J Arnold Arbor. 1985b; 66: 243–273.

21. McLoughlin S. The breakup history of Gondwana and its impact on pre-Cenozoic floristic provincialism. Aust J Bot. 2001; 49(3): 271–300.

22. Ezcurra MD, Agnolín FL. A new global palaeobiogeographical model for the late Mesozoic and early Tertiary. Syst Biol. 2012; 61(4), 553–66.

23. Kastens K, Bonatti E, Caress D. The Vema transverse ridge (central Atlantic). Mar Geophys Res. 1998; 20: 533–556.

24. Lawver L, Gahagan L. Evolution of Cenozoic seaways in the circum-Antarctic region. Palaeogeogr Palaeoclimatol Palaeoecol. 2003; 198: 11–37.

25. Markwick PJ, Valdes PJ. Palaeo-digital elevation models for use as boundary conditions in coupled ocean–atmosphere GCM experiments: a Maastrichtian (late Cretaceous) example. Palaeogeo Palaeoclimatol Palaeoecol. 2004; 213(1-2): 37–63.

26. Audley-Charles MG. Tectonics of the New Guinea area. Annu Rev Earth Planet Sci Lett. 1991; 19: 17–41.

27. Metcalfe I. Palaeozoic and Mesozoic geological evolution of the SE Asian region: multidisciplinary constraints and implications for biogeography. In: Hall R., Holloway JD. Biogeography and geological evolution of SE Asia. Leiden: Backhuys Publishers; 1998. pp. 25–41.

28. Neall VE, Trewick SA. The age and origin of the Pacific islands: A geological overview. Philos Trans R Soc B. 2008; 363(1508): 3293–3308.

29. Mann P. Overview of plate tectonic history and its unresolved tectonic problems. In: Bundschuh J, Alvaro GE. Central America: Geology, Resources and Hazards. London: Taylor & Francis Inc; 2007. pp. 205–241.

30. Nougier J, Cantagrel JM, Karche JP. The Comores archipelago in the western Indian Ocean: Volcanology, geochronology and geodynamic setting. J Afr Earth Sci. 1986; 5(2): 135–145.

31. Rocha S, Carretero MA, Harris DJ. Mitochondrial DNA sequence data suggests two independent colonizations of the Comoros archipelago by Chameleons of the genus Furcifer Belg J Zool. 2005; 135: 39–42.
