# Supplementary material for: The Gondwana Breakup and the History of the Atlantic and Indian Oceans Unveils Two New Clades for Early Neobatrachian Diversification
Source: PLoS One. 2015 Nov 30;10(11):e0143926. doi: 10.1371/journal.pone.0143926 (PMC4664409; doi:10.1371/journal.pone.0143926)
Supplement: S1 Table — GenBank accession numbers for each species analysed. (DOC) [file pone.0143926.s006.doc]

Table S1: Table with genus and species respective followed by GenBank accession numbers used for phylogenetic reconstruction and divergence time inference. Shaded genus are out-group selected. The list of genus is alphabetically sorted. c-myc (*myelocytomatosis* *exon* 2 e 3), cyt-b (*cytochrome* b), H3A (*histone* 3a), ND1 (NADH *dehydrogenase subunit* 1), POMC (*proopiomelanocortin*), RAG-1 (*recombination activating gene* 1), RHOD (*rhodopsin*), SIA (*seventh in absentia*), TyrPrecur (*tyrodinase precursor*), 12S,16S e 28S. *Names redetermined. See Blotto et al. (2013), Fouquet et al. (2013), Faivovich et al. (2014).

| **Gênero** | **cmyc 2** | **cmyc 3** | **cytb** | **H3A** | **ND1** | **POMC** | **RAG1** | **RHOD** | **SIA** | **TyrPrecur** | **12S** | **16S** | **28S** |
| --- | --- | --- | --- | --- | --- | --- | --- | --- | --- | --- | --- | --- | --- |
| *Bombina* |  |  | Bombina maxima (NC011049) | Bombina bombina (DQ284275) | Bombina maxima (NC011049) | Bombina orientalis (AY692246) | Bombina orientalis (AY364207) | Bombina orientalis (DQ284032) | Bombina maxima (DQ282865) |  | Bombina maxima (NC011049) | Bombina maxima (NC011049) | Bombina orientalis (DQ283741) |
| *Pelobates* |  |  | Pelobates cultripes (NC008144) | Pelobates fuscus (DQ284159) | Pelobates cultripes (NC008144) | Pelobates fuscus fuscus (HM998966) | Pelobates cultripes (AY364201) | Pelobates fuscus (DQ283826) |  | Pelobates cultripes (DQ347131) | Pelobates cultripes (NC008144) | Pelobates cultripes (NC008144) | Pelobates fuscus (DQ283511) |
| *Pipa* |  |  | Pipa pipa (GQ244477) | Pipa carvalhoi (DQ284277) | Pipa pipa (GQ244477) | Pipa carvalhoi (HM998963) | Pipa pipa (AY364204) | Pipa pipa (DQ283781) | Pipa pipa (DQ282660) | Pipa pipa (DQ347133) | Pipa pipa (GQ244477) | Pipa pipa (GQ244477) | Pipa carvalhoi (DQ283613) |
| *Xenopus* | Xenopus laevis (AY819160) | Xenopus laevis (AY819240) | Xenopus victorianus (NC018775) | Xenopus gilli (DQ284418) | Xenopus victorianus (NC018775) | Xenopus laevis (NM001087369) | Xenopus muelleri (AY874356) | Xenopus victorianus (NM001087048) |  | Xenopus laevis (AB108529) | Xenopus victorianus (NC018775) | Xenopus victorianus (NC018775) | Xenopus gilli (DQ283750) |
| *Allophryne* | Allophryne ruthveni (AY819162) | Allophryne ruthveni (AY819242) | Allophryne ruthveni (AY843786) |  |  | Allophryne ruthveni (AY819077) | Allophryne ruthveni (AY844361) | Allophryne ruthveni (AY844538) | Allophryne ruthveni (AY844766) |  | Allophryne ruthveni (JQ436703) | Allophryne ruthveni (JQ436701) |  |
| *Alsodes* |  |  | Alsodes neuquensis *(AY843787) | Alsodes neuquensis *(DQ284118) |  |  | Alsodes neuquensis *(AY844362) | Alsodes neuquensis *(AY844539) | Alsodes neuquensis *(AY844767) |  | Alsodes verrucosus (JX204191) | Alsodes verrucosus (JX204191) | Alsodes neuquensis *(AY844197) |
| *Eupsophus* |  |  | Eupsophus calcaratus *(HQ710989) | Eupsophus calcaratus *(DQ284120) |  |  |  | Eupsophus calcaratus *(AY844560) | Eupsophus calcaratus *(AY844786) | Eupsophus calcaratus *(AY844036) | Eupsophus vertebralis (JX204221) | Eupsophus vertebralis (JX204221) | Eupsophus calcaratus *(AY844214) |
| *Limnomedusa* |  |  | Limnomedusa macroglossa (AY843935) | Limnomedusa macroglossa (DQ284127) |  |  | Limnomedusa macroglossa (AY844471) |  | Limnomedusa macroglossa (AY844891) | Limnomedusa macroglossa (AY844128) | Limnomedusa macroglossa (AY843689) | Limnomedusa macroglossa (AY843689) |  |
| *Allobates* | Allobates trilineatus (AY819173) | Allobates trilineatus (AY819253) | Allobates zaparo (HQ290580) | Allobates femoralis (DQ284074) | Allobates zaparo (HQ291003) | Allobates kingsburyi (HQ290841) | Allobates talamancae (AY844373) | Allobates zaparo (DQ503192) | Allobates brunneus (DQ503098) | Allobates femoralis (DQ503156) | Allobates zaparo (HQ291003) | Allobates femoralis (JQ436719) | Allobates zaparo (DQ502955) |
| *Aromobates* |  |  | Aromobates saltuensis (HQ290548) | Aromobates nocturnus (DQ502359) | Aromobates saltuensis (HQ290970) | Aromobates saltuensis (HQ290848) | Aromobates saltuensis (DQ503406) | Aromobates nocturnus (DQ503243) | Aromobates nocturnus (DQ503107) | Aromobates saltuensis (HQ290908) | Aromobates saltuensis (JX035996) | Aromobates saltuensis (JX035996) | Aromobates nocturnus (DQ502996) |
| *Scotobleps* |  |  |  | Scotobleps gabonicus (DQ284353) |  |  |  | Scotobleps gabonicus (DQ283988) | Scotobleps gabonicus (DQ282834) |  | Scotobleps gabonicus (DQ283367) | Scotobleps gabonicus (DQ283367) | Scotobleps gabonicus (DQ283686) |
| *Arthroleptis* |  |  |  | Arthroleptis sylvaticus (DQ284131) |  |  |  | Arthroleptis sylvaticus (DQ283801) | Arthroleptis sylvaticus (DQ282666) | Arthroleptis sylvaticus (DQ282912) | Arthroleptis sylvaticus (DQ283077) | Arthroleptis sylvaticus (DQ283077) | Arthroleptis sylvaticus (DQ283482) |
| *Atelognathus* |  |  | Atelognathus patagonicus (AY843793) |  |  |  | Atelognathus patagonicus (AY844368) | Atelognathus patagonicus (AY844545) | Atelognathus patagonicus (AY844773) | Atelognathus patagonicus (AY844027) | Atelognathus patagonicus (AY843571) | Atelognathus patagonicus (AY843571) | Atelognathus patagonicus (AY844203) |
| *Batrachyla* |  |  | Batrachyla leptopus (AY843794) | Batrachyla leptopus (DQ284119) | Batrachyla taeniata (FJ882753) |  | Batrachyla leptopus (AY844369) | Batrachyla leptopus (AY844546) | Batrachyla leptopus (AY844774) | Batrachyla leptopus (AY844028) | Batrachyla leptopus (AY843572) | Batrachyla leptopus (AY843572) | Batrachyla leptopus (AY844204) |
| *Hylorina* |  |  | Hylorina sylvatica (AY389143) |  |  |  |  |  |  |  | Hylorina sylvatica (JX204222) | Hylorina sylvatica (JX204222) |  |
| *Brachycephalus* | Brachycephalus ephippium (GQ345145) | Brachycephalus ephippium (GQ345162) | Brachycephalus ephippium (HM216363) |  | Brachycephalus ephippium (GQ345243) | Brachycephalus ephippium (GQ345256) | Brachycephalus ephippium (GQ345275) | Brachycephalus ephippium (DQ283806) | Brachycephalus ephippium (DQ282672) | Brachycephalus didactylus (JX267681) | Brachycephalus ephippium (DQ283091) | Brachycephalus ephippium (DQ283091) | Brachycephalus ephippium (DQ283492) |
| *Ischnocnema* | Ischnocnema aff. ramagii (EU025676) | Ischnocnema aff. ramagii (EU025676) | Ischnocnema parva (HQ435717) | Ischnocnema juipoca (DQ284143) | Ischnocnema parva (GQ345244) | Ischnocnema guentheri (GQ345257) | Ischnocnema guentheri (GQ345276) | Ischnocnema juipoca (DQ283809) | Ischnocnema guentheri (GQ345308) | Ischnocnema parva (EF493509) | Ischnocnema vizottoi (JX267458) | Ischnocnema venancioi (JX267382) | Ischnocnema juipoca (DQ283495) |
| *Breviceps* |  |  | Breviceps mossambicus (FJ998375) | Breviceps mossambicus (DQ284397) |  |  | Breviceps mossambicus (EF018056) | Breviceps mossambicus (DQ284023) |  | Breviceps mossambicus (DQ283013) | Breviceps mossambicus (DQ283155) | Breviceps mossambicus (DQ283155) | Breviceps mossambicus (DQ283546) |
| *Callulina* |  |  | Callulina kisiwamsitu (FJ998380) | Callulina kisiwamsitu (DQ284406) |  |  | Callulina kreffti (DQ347281) | Callulina kreffti (DQ347400) | Callulina kisiwamsitu (DQ282884) | Callulina kisiwamsitu (DQ283021) | Callulina kisiwamsitu (DQ283429) | Callulina kisiwamsitu (DQ283429) | Callulina kisiwamsitu (DQ283737) |
| *Adenomus* |  |  |  |  | Adenomus kelaartii (FJ882780) |  | Adenomus kelaartii (EF107284) |  |  |  | Adenomus kelaartii (FJ882780) | Adenomus kelaartii (FJ882780) |  |
| *Amietophrynus* |  |  | Amietophrynus pardalis (AF210079) | Amietophrynus maculatus (DQ284374) | Amietophrynus regularis (DQ629609) | Amietophrynus steindachneri (DQ158329) | Amietophrynus regularis (AY323763) | Amietophrynus gutturalis (DQ284035) | Amietophrynus brauni (DQ282873) | Amietophrynus brauni (DQ283011) | Amietophrynus xeros (DQ158499) | Amietophrynus xeros (DQ158499) | Amietophrynus latifrons (DQ283665) |
| *Anaxyrus* | Anaxyrus americanus (GQ442777) |  | Anaxyrus punctatus (JQ947916) | Anaxyrus woodhousii (DQ284222) | Anaxyrus exsul (AF004524) | Anaxyrus punctatus (JQ947872) | Anaxyrus debilis (DQ158371) | Anaxyrus hemiophrys (FJ004272) | Anaxyrus woodhousii (DQ282733) |  | Anaxyrus woodhousii (DQ158498) | Anaxyrus woodhousii (DQ158498) | Anaxyrus quercicus (DQ283544) |
| *Ansonia* |  |  |  |  | Ansonia muelleri (JN314736) |  |  | Ansonia longidigita (DQ283968) | Ansonia longidigita (DQ282817) |  | Ansonia longidigita (FJ882796) | Ansonia longidigita (FJ882796) |  |
| *Atelopus* | Atelopus peruensis (AY819163) | Atelopus peruensis (AY819243) | Atelopus varius (EF494952) | Atelopus flavescens (DQ284282) | Atelopus peruensis (AY819459) | Atelopus peruensis (DQ158261) | Atelopus peruensis (DQ158345) | Atelopus flavescens (DQ283928) | Atelopus spumarius (DQ282781) | Atelopus flavescens (EF364359) | Atelopus peruensis (DQ158419) | Atelopus peruensis (DQ158419) |  |
| *Bufo* |  |  | Bufo gargarizans (L10972) | Bufo gargarizans andrewsi (DQ284260) | Bufo gargarizans (AF004535) | Bufo gargarizans andrewsi (DQ158269) | Bufo bufo (AY323762) | Bufo gargarizans andrewsi (DQ283905) | Bufo gargarizans andrewsi (DQ282763) |  | Bufo bufo (DQ158438) | Bufo bufo (DQ158438) | Bufo gargarizans (DQ283599) |
| *Capensibufo* |  |  |  |  |  |  |  |  |  |  | Capensibufo rosei (AF220864) | Capensibufo rosei (AF220911) |  |
| *Churamiti* |  |  |  |  | Churamiti maridadi (FJ882770) |  |  |  |  |  | Churamiti maridadi (FJ882769) | Churamiti maridadi (FJ882769) |  |
| *Amazophrynella* | Amazophrynella minuta (AY819166) | Amazophrynella minuta (AY819246) | Amazophrynella minuta (AY843804) | Amazophrynella minuta (DQ284096) | Amazophrynella minuta (AY819462) | Amazophrynella minuta (DQ158262) |  | Amazophrynella minuta (AY844555) |  | Amazophrynella minuta (EF364362) | Amazophrynella minuta (DQ158420) | Amazophrynella minuta (DQ158420) |  |
| *Didynamipus* |  |  |  |  |  |  |  |  |  |  | Didynamipus sjostedti (AY325991) | Didynamipus sjostedti (AY325991) |  |
| *Duttaphrynus* |  |  | Duttaphrynus melanostictus (AF249082) | Duttaphrynus melanostictus (DQ284324) | Duttaphrynus melanostictus (AY458592) | Duttaphrynus melanostictus (DQ158317) | Duttaphrynus melanostictus (AY364197) | Duttaphrynus melanostictus (DQ283967) | Duttaphrynus melanostictus (DQ282815) |  | Duttaphrynus melanostictus (NC_005794) | Duttaphrynus melanostictus (NC_005794) | Duttaphrynus melanostictus (DQ283658) |
| *Epidalea* |  |  | Epidalea calamita (L10963) |  | Epidalea calamita (DQ629607) |  |  |  |  |  | Epidalea calamita (FJ882809) | Epidalea calamita (FJ882809) |  |
| *Ghatophryne* |  |  |  |  |  |  |  |  |  |  | Ghatophryne ornata (FJ882797) | Ghatophryne ornata (FJ882797) |  |
| *Incilius* | Incilius alvarius (AY819164) | Incilius alvarius (AY819244) | Incilius nebulifer (HQ290525) | Incilius coniferus (DQ284204) | Incilius nebulifer (HQ290945) | Incilius occidentalis (JQ947856) | Incilius coniferus (DQ158368) | Incilius coniferus (DQ283860) | Incilius coniferus (DQ282719) | Incilius nebulifer (HQ290885) | Incilius alvarius (DQ158425) | Incilius alvarius (DQ158425) | Incilius coniferus (DQ283556) |
| *Ingerophrynus* |  |  |  | Ingerophrynus galeatus (DQ284362) |  | Ingerophrynus macrotis (DQ158309) | Ingerophrynus galeatus (DQ158374) | Ingerophrynus galeatus (DQ283995) | Ingerophrynus galeatus (DQ282839) | Ingerophrynus galeatus (DQ282987) | Ingerophrynus macrotis (DQ158468) | Ingerophrynus macrotis (DQ158468) | Ingerophrynus divergens (DQ283540) |
| *Leptophryne* |  |  |  |  | Leptophryne borbonica (FJ882799) |  | Leptophryne borbonica (EF107287) |  |  |  | Leptophryne borbonica (FJ882799) | Leptophryne borbonica (FJ882799) |  |
| *Melanophryniscus* | Melanophryniscus sp. (AY819167) | Melanophryniscus sp. (AY819247) | Melanophryniscus klappenbachi (DQ502444) | Melanophryniscus klappenbachi (DQ284060) | Melanophryniscus sp. (AY819463) | Melanophryniscus stelzneri (DQ158263) | Melanophryniscus klappenbachi (AY844478) | Melanophryniscus klappenbachi (DQ283765) | Melanophryniscus klappenbachi (AY844899) |  | Melanophryniscus klappenbachi (AY843699) | Melanophryniscus klappenbachi (AY843699) | Melanophryniscus klappenbachi (AY844306) |
| *Mertensophryne* |  |  |  |  |  |  | Mertensophryne micranotis (EF107330) |  |  |  | Mertensophryne lindneri (FJ882847) | Mertensophryne lindneri (FJ882847) |  |
| *Nannophryne* |  |  |  |  |  | Nannophryne variegata (DQ158335) | Nannophryne cophotis (DQ158369) |  |  |  | Nannophryne variegata (DQ158494) | Nannophryne variegata (DQ158494) |  |
| *Nectophryne* |  |  |  | Nectophryne afra (DQ284347) |  |  |  | Nectophryne afra (DQ283981) | Nectophryne batesii (DQ282721) | Nectophryne afra (DQ282977) | Nectophryne batesii (DQ283169) | Nectophryne batesii (DQ283169) | Nectophryne batesii (DQ283559) |
| *Nectophrynoides* |  |  |  | Nectophrynoides tornieri (DQ284394) | Nectophrynoides viviparus (FJ882816) |  | Nectophrynoides tornieri (EF107329) | Nectophrynoides tornieri (DQ284018) | Nectophrynoides tornieri (DQ282870) |  | Nectophrynoides tornieri (DQ283413) | Nectophrynoides tornieri (DQ283413) | Nectophrynoides tornieri (DQ283723) |
| *Nimbaphrynoides* |  |  | Nimbaphrynoides liberiensis (GU322859) |  |  |  |  |  |  |  | Nimbaphrynoides occidentalis (GU322836) | Nimbaphrynoides occidentalis (GU322848) |  |
| *Oreophrynella* |  |  |  |  |  | Oreophrynella sp. (DQ158264) | Oreophrynella sp. (DQ158348) |  |  |  | Oreophrynella sp. (DQ158422) | Oreophrynella sp. (DQ158422) |  |
| *Osornophryne* | Osornophryne guacamayo (AY819168) | Osornophryne guacamayo (AY819248) |  |  | Osornophryne guacamayo (AY819464) | Osornophryne guacamayo (AY819083) |  |  |  |  | Osornophryne antisana (JX418268) | Osornophryne simpsoni (JX412037) |  |
| *Pedostibes* |  |  |  | Pedostibes hosii (DQ284202) | Pedostibes hosii (FJ882804) |  | Pedostibes hosii (EF107286) | Pedostibes hosii (DQ283859) | Pedostibes hosii (DQ282717) |  | Pedostibes hosii (DQ283164) | Pedostibes hosii (DQ283164) |  |
| *Pelophryne* |  |  |  |  | Pelophryne signata (FJ882801) |  |  |  |  |  | Pelophryne signata (FJ882801) | Pelophryne signata (FJ882801) |  |
| *Peltophryne* |  |  | Peltophryne fustiger (AF361698) |  |  | Peltophryne lemur (DQ158306) | Peltophryne peltocephalus (JF342388) |  |  |  | Peltophryne lemur (DQ283273) | Peltophryne lemur (DQ283273) |  |
| *Phrynoidis* |  |  |  | Phrynoidis asper (DQ284188) | Phrynoidis juxtaspera (FJ882805) | Phrynoidis asper (DQ158273) | Phrynoidis juxtaspera (DQ158385) | Phrynoidis asper (DQ283848) | Phrynoidis asper (DQ282704) | Phrynoidis asper (DQ282939) | Phrynoidis asper (DQ158432) | Phrynoidis asper (DQ158432) | Phrynoidis asper (DQ283539) |
| *Poyntonophrynus* |  |  |  |  |  |  |  |  |  |  | Poyntonophrynus vertebralis (AF220860) | Poyntonophrynus dombensis (AF220907) |  |
| *Pseudepidalea* |  |  | Pseudepidalea viridis (L10982) | Pseudepidalea viridis (DQ284297) | Pseudepidalea viridis (AF004525) |  |  | Pseudepidalea viridis (DQ283940) | Pseudepidalea viridis (DQ282791) |  | Pseudepidalea viridis (JX439781) | Pseudepidalea shaartusiensis (HQ843047) | Pseudepidalea viridis (DQ283630) |
| *Rhaebo* |  |  | Rhaebo haematiticus (HM563930) | Rhaebo guttatus (DQ284361) |  | Rhaebo nasicus (DQ158319) | Rhaebo glaberrimus (DQ158377) | Rhaebo guttatus (DQ283994) | Rhaebo haematiticus (DQ282720) | Rhaebo guttatus (EF364361) | Rhaebo glaberrimus (DQ158454) | Rhaebo glaberrimus (DQ158454) | Rhaebo haematiticus (DQ283557) |
| *Rhinella* | Rhinella arenarum (JN594525) | Rhinella margaritifera (AY819245) | Rhinella margaritifera ( JX298409) | Rhinella paracnemis (DQ284102) | Rhinella margaritifera (AY819461) | Rhinella marina (DQ158315) | Rhinella nesiotes (DQ158397) | Rhinella arenarum (AY844547) | Rhinella arenarum (AY844775) | Rhinella margaritifera (EF364343) | Rhinella veraguensis (DQ158496) | Rhinella veraguensis (DQ158496) | Rhinella arenarum (AY844205) |
| *Vandijkophrynus* |  |  |  |  | Vandijkophrynus robinsoni (GU183857) |  |  |  |  |  | Vandijkophrynus robinsoni (GU183857) | Vandijkophrynus robinsoni (GU183857) |  |
| *Schismaderma* |  |  |  | Schismaderma carens (DQ284403) |  | Schismaderma carens (DQ158266) | Schismaderma carens (DQ158350) | Schismaderma carens (DQ284027) | Schismaderma carens (DQ282882) |  | Schismaderma carens (DQ283425) | Schismaderma carens (DQ283425) | Schismaderma carens (DQ283734) |
| *Werneria* |  |  |  | Werneria mertensiana (DQ284338) |  |  |  | Werneria mertensiana (DQ283974) | Werneria mertensiana (DQ282824) |  | Werneria mertensiana (DQ283348) | Werneria mertensiana (DQ283348) | Werneria mertensiana (DQ283672) |
| *Wolterstorffina* |  |  |  | Wolterstorffina parvipalmata (DQ284334) | Wolterstorffina parvipalmata (FJ882818) |  |  | Wolterstorffina parvipalmata (DQ283972) | Wolterstorffina parvipalmata (DQ282822) |  | Wolterstorffina parvipalmata (DQ283346) | Wolterstorffina parvipalmata (DQ283346) | Wolterstorffina parvipalmata (DQ283668) |
| *Calyptocephalella* |  |  | Calyptocephallela gayi (JX298415) |  |  |  |  |  |  | Calyptocephallela gayi (JX298244) | Calyptocephallela gayi (FJ882733) | Calyptocephallela gayi (FJ882733) |  |
| *Telmatobufo* |  |  |  | Telmatobufo venustus (DQ284321) |  |  |  | Telmatobufo venustus (DQ283964) | Telmatobufo venustus (DQ282814) |  | Telmatobufo australis (AF144716) | Telmatobufo bullocki (GQ994987) | Telmatobufo venustus (DQ283655) |
| *Hyalinobatrachium* | Hyalinobatrachium colymbiphyllum (AY819172) | Hyalinobatrachium colymbiphyllum (AY819252) |  | Hyalinobatrachium fleischmanni (DQ284426) | Hyalinobatrachium colymbiphyllum (AY819468) | Hyalinobatrachium tatayoi (EU663234) | Hyalinobatrachium valerioi (EU663519) | Hyalinobatrachium fleischmanni (DQ284043) | Hyalinobatrachium eurygnathum (AY844793) |  | Hyalinobatrachium fleischmanni (DQ283453) | Hyalinobatrachium fleischmanni (DQ283453) | Hyalinobatrachium eurygnathum (AY844217) |
| *Nymphargus* | Nymphargus griffithsi (AY819171) | Nymphargus griffithsi (AY819251) | Nymphargus bejaranoi (AY843798) | Nymphargus bejaranoi (DQ284066) | Nymphargus griffithsi (AY819467) | Nymphargus wileyi (EU663248) | Nymphargus bejaranoi (AY844372) | Nymphargus bejaranoi (AY844549) | Nymphargus bejaranoi (AY844777) | Nymphargus bejaranoi (AY844029) | Centrolene grandisonae (HQ290946) | Centrolene grandisonae (HQ290946) | Nymphargus bejaranoi (AY844208) |
| *Ceratobatrachus* |  |  |  | Ceratobatrachus guentheri (DQ284409) | Ceratobatrachus guentheri (AY948752) |  | Ceratobatrachus guentheri (DQ347272) | Ceratobatrachus guentheri (DQ284031) | Ceratobatrachus guentheri (DQ282886) | Ceratobatrachus guentheri (DQ283024) | Ceratobatrachus guentheri (DQ283197) | Ceratobatrachus guentheri (DQ283197) | Ceratobatrachus guentheri (DQ283579) |
| *Batrachylodes* |  |  |  | Batrachylodes vertebralis (DQ284242) |  |  | Batrachylodes sp. (DQ347228) | Batrachylodes vertebralis (DQ283891) | Batrachylodes vertebralis (DQ282753) | Batrachylodes sp. (DQ347136) | Batrachylodes vertebralis (DQ283210) | Batrachylodes vertebralis (DQ283210) | Batrachylodes vertebralis (DQ283586) |
| *Ceratophrys* | Ceratophrys cornuta (AY819176) | Ceratophrys cornuta (AY819255) | Ceratophrys cornuta (HQ290527) |  | Ceratophrys cornuta (HQ290947) | Ceratophrys cornuta (HQ290827) | Ceratophrys ornata (AY364218) | Ceratophrys cranwelli *(AY364399) |  | Ceratophrys cornuta (HQ290887) | Ceratophrys cranwelli  *(DQ347035) | Ceratophrys cranwelli *(DQ347035) | Ceratophrys cranwelli (AY844207) |
| *Chacophrys* |  |  |  |  |  |  |  |  |  |  | Chacophrys pierottii (DQ283328) | Chacophrys pierottii (DQ283328) |  |
| *Lepidobatrachus* | Lepidobatrachus laevis (AY819179) | Lepidobatrachus laevis (AY819258) |  | Lepidobatrachus laevis (DQ284191) |  |  | Lepidobatrachus laevis (DQ679270) | Lepidobatrachus laevis (DQ283851) | Lepidobatrachus laevis (DQ282707) |  | Lepidobatrachus laevis (DQ283152) | Lepidobatrachus laevis (DQ283152) | Lepidobatrachus laevis (DQ283543) |
| *Ceuthomantis* | Ceuthomantis smaragdinus (GQ345154) | Ceuthomantis smaragdinus (GQ345169) | Ceuthomantis smaragdinus (GQ345208) |  | Ceuthomantis smaragdinus (GQ345253) | Ceuthomantis smaragdinus (GQ345267) | Ceuthomantis smaragdinus (GQ345287) | Ceuthomantis smaragdinus (GQ345305) | Ceuthomantis smaragdinus (GQ345318) |  | Ceuthomantis smaragdinus (GQ345132) | Ceuthomantis smaragdinus (GQ345132) | Ceuthomantis smaragdinus (GQ345142) |
| *Conraua* |  |  | Conraua goliath (AY014385) | Conraua robusta (DQ284337) |  |  | Conraua crassipes (DQ019498) | Conraua robusta (DQ283973) | Conraua robusta (DQ282823) | Conraua robusta (DQ282972) | Conraua robusta (DQ283347) | Conraua robusta (DQ283347) | Conraua robusta (DQ283671) |
| *Craugastor* | Craugastor bransfordii (AY211321) | Craugastor bransfordii (AY211321) | Craugastor andi (EF629473) | Craugastor alfredi (DQ284318) | Craugastor fitzingeri (GQ345245) | Craugastor podiciferus (GQ345258) | Craugastor podiciferus (GQ345277) | Craugastor punctariolus (DQ283862) | Craugastor rhodopis (DQ282808) | Craugastor uno (GU576489) | Craugastor rhodopis (DQ283317) | Craugastor rhodopis (DQ283317) | Craugastor ranoides (DQ283505) |
| *Haddadus* | Haddadus binotatus (GQ345147) | Haddadus binotatus (GQ345165) | Haddadus binotatus (GQ345198) | Haddadus binotatus (DQ284142) |  | Haddadus binotatus (GQ345259) | Haddadus binotatus (GQ345293) | Haddadus binotatus (DQ283807) | Haddadus binotatus (GQ345309) | Haddadus binotatus (JX267685) | Haddadus binotatus (JX267346) | Haddadus binotatus (JX267346) | Haddadus binotatus (DQ283493) |
| *Cycloramphus* |  |  | Cycloramphus acangatan (FJ685663) | Cycloramphus boraceiensis (DQ284147) |  |  | Cycloramphus acangatan (HQ634170) | Cycloramphus boraceiensis (DQ283813) | Cycloramphus boraceiensis (DQ282675) | Cycloramphus boraceiensis (DQ282924) | Cycloramphus boraceiensis (DQ283097) | Cycloramphus boraceiensis (DQ283097) | Cycloramphus boraceiensis (DQ283498) |
| *Thoropa* | Thoropa taophora (GQ345158) | Thoropa taophora (GQ345172) | Thoropa miliaris (FJ685662) |  | Thoropa taophora (GQ345254) | Thoropa taophora (GQ345271) | Thoropa taophora (GQ345288) | Thoropa taophora (GQ345307) | Thoropa taophora (GQ345320) |  | Thoropa taophora *(DQ283331) | Thoropa taophora *(DQ283331) | Thoropa taophora (GQ345143) |
| *Zachaenus* |  |  |  |  |  |  |  |  |  |  | Zachaenus parvulus (JQ966107) | Zachaenus parvulus (JQ966107) |  |
| *Dendrobates* |  |  | Dendrobates auratus (HQ290557) | Dendrobates auratus (DQ284072) | Dendrobates leucomelas (HQ290987) | Dendrobates truncatus (HQ290869) | Dendrobates azureus (DQ503338) | Dendrobates tinctorius (DQ503229) | Dendrobates tinctorius (DQ503133) | Dendrobates auratus (AY844032) | Dendrobates leucomelas (HQ290987) | Dendrobates leucomelas (HQ290987) | Dendrobates azureus (DQ503031) |
| *Silverstoneia* |  |  | Silverstoneia flotator (HQ290537) | Silverstoneia nubicola (DQ502330) | Silverstoneia flotator (HQ290957) | Silverstoneia nubicola (HQ290844) | Silverstoneia flotator (DQ503362) | Silverstoneia nubicola (DQ503245) | Silverstoneia nubicola (DQ503082) | Silverstoneia flotator (HQ290897) | Silverstoneia flotator (DQ502162) | Silverstoneia flotator (DQ502162) | Silverstoneia nubicola (DQ502981) |
| *Limnonectes* |  |  | Limnonectes kuhlii (FJ349553) | Limnonectes kuhlii (DQ284355) |  | Limnonectes kuhlii (GU934419) | Limnonectes kuhlii (DQ347232) | Limnonectes kuhlii (DQ283990) | Limnonectes kuhlii (DQ282835) | Limnonectes kuhlii (DQ282982) | Limnonectes kuhlii (DQ283370) | Limnonectes kuhlii (DQ283370) | Limnonectes kuhlii (DQ283688) |
| *Nannophrys* |  |  | Nannophrys ceylonensis (AF249093) |  | Nannophrys ceylonensis (AY948731) |  | Nannophrys ceylonensis (GQ204610) | Nannophrys ceylonensis (AF249112) |  | Nannophrys ceylonensis (AF249175) | Nannophrys ceylonensis (DQ346975) | Nannophrys ceylonensis (DQ346975) |  |
| *Adelophryne* | Adelophryne gutturosa (GQ345149) | Adelophryne gutturosa (GQ345167) | Adelophryne gutturosa (GQ345201) |  | Adelophryne gutturosa (GQ345247) | Adelophryne gutturosa (GQ345262) | Adelophryne gutturosa (GQ345280) | Adelophryne gutturosa (GQ345302) | Adelophryne gutturosa (GQ345312) | Adelophryne baturitensis (JX267679) | Adelophryne gutturosa (EU186679) | Adelophryne gutturosa (EU186679) | Adelophryne gutturosa (GQ345136) |
| *Eleutherodactylus* | Eleutherodactylus pantoni (AY211282) | Eleutherodactylus cooki (GQ345166) | Eleutherodactylus varleyi (JN015150) | Eleutherodactylus nitidus (DQ284316) | Eleutherodactylus planirostris (GQ345246) | Eleutherodactylus planirostris (HQ831960) | Eleutherodactylus cooki (GQ345294) | Eleutherodactylus portoricensis (HQ823686) | Eleutherodactylus marnockii (DQ282677) | Eleutherodactylus planirostris (DQ282929 ) | Eleutherodactylus varleyi (JF947374) | Eleutherodactylus varleyi (JF947368) | Eleutherodactylus marnockii (DQ283502) |
| *Hadromophryne* |  |  |  |  |  |  |  |  |  |  | Hadromophryne natalensis (X86237) | Hadromophryne natalensis (AY176692) |  |
| *Heleophryne* |  |  | Heleophryne purcelli (AY843812) | Heleophryne regis (DQ284161) | Heleophryne purcelli (AY948755) |  | Heleophryne purcelli (AY364221) | Heleophryne regis (DQ283828) | Heleophryne regis (DQ282684) |  | Heleophryne purcelli (AY326072) | Heleophryne purcelli (X86271) | Heleophryne purcelli (AY844216) |
| *Flectonotus* | Flectonotus fitzgeraldi (AY819189) | Flectonotus fitzgeraldi (AY819265) | Flectonotus sp. CFBH 5720 (AY843809) |  | Flectonotus fitzgeraldi (AY819486) | Flectonotus fitzgeraldi (AY819104) | Flectonotus fitzgeraldi (DQ679274) | Flectonotus sp. (AY844562) | Flectonotus sp. (AY844788) | Flectonotus sp. (AY844038) | Flectonotus pygmaeus (DQ679232) | Flectonotus pygmaeus (DQ679382) | Flectonotus sp. (AY844215) |
| *Hemiphractus* | Hemiphractus proboscideus (AY819192) | Hemiphractus proboscideus (AY819266) | Hemiphractus helioi (AY843813) | Hemiphractus helioi (DQ284084) | Hemiphractus scutatus (DQ679371) | Hemiphractus bubalus (DQ679335) | Hemiphractus proboscideus (DQ679304) | Hemiphractus helioi (AY844566) | Hemiphractus helioi (AY844792) |  | Hemiphractus helioi (AY843594) | Hemiphractus helioi (AY843594) | Hemiphractus bubalus (GQ345134) |
| *Litoria* | Litoria aurea (AY819233) | Litoria caerulea (AY819308) | Litoria infrafrenata (AY843940) | Litoria meiriana (DQ284125) | Litoria caerulea (GQ366301) | Litoria aurea (GQ366037) | Litoria caerulea (AY323767) | Litoria caerulea (AY323751) | Litoria lesueurii (DQ282747) | Litoria aurea (AY844130) | Litoria aurea (AY843691) | Litoria aurea (AY843691) | Litoria infrafrenata (AY844304) |
| *Phyllomedusa* | Phyllomedusa tomopterna (AY819239) | Phyllomedusa tomopterna (AY819313) | Phyllomedusa centralis (GQ365948) |  | Phyllomedusa tomopterna (GQ366337) | Phyllomedusa sauvagii (GQ366065) | Phyllomedusa tomopterna (EF174319) | Phyllomedusa bicolor (AY844710) | Phyllomedusa tarsius (AY844918) | Phyllomedusa hypochondrialis (AY844153) | Phyllomedusa ayeaye (GQ366244) | Phyllomedusa ayeaye (GQ366244) | Phyllomedusa tetraploidea (AY844327) |
| *Scinax* | Scinax catharinae (AY819225) | Scinax crospedospilus (AY819300) | Scinax nebulosus (AF549373) | Scinax ruber (DQ284045) | Scinax perpusillus (JN100015) | Scinax staufferi (GQ366071) | Scinax acuminatus (AY844515) | Scinax catharinae (AY844742) | Scinax uruguayus (AY844884) | Scinax fuscovarius (AY844179) | Scinax fuscovarius (AY843758) | Scinax fuscovarius (AY843758) | Scinax uruguayus (AY844299) |
| *Crossodactylus* |  |  | Crossodactylus schmidti (HQ290528) | Crossodactylus schmidti (DQ284050) | Crossodactylus schmidti (HQ290948) | Crossodactylus schmidti (HQ290828) | Crossodactylus schmidti (DQ503298) | Crossodactylus schmidti (AY844552) | Crossodactylus schmidti (AY844780) | Crossodactylus schmidti (AY844031) | Crossodactylus schmidti (AY843579) | Crossodactylus schmidti (AY843579) | Crossodactylus schmidti (AY844210) |
| *Hylodes* | Hylodes nasus (GQ345159) | Hylodes nasus (GQ345173) | Hylodes phyllodes (DQ502606) | Hylodes phyllodes (DQ284146) | Hylodes nasus (GQ345255) | Hylodes nasus (GQ345272) | Hylodes nasus (GQ345289) | Hylodes phyllodes (DQ503253) | Hylodes phyllodes (DQ503119) | Hylodes phyllodes (DQ282923) | Hylodes phyllodes (DQ502171) | Hylodes phyllodes (DQ502171) | Hylodes phyllodes (DQ503009) |
| *Hyperolius* | Hyperolius mitchelli (HM772447) | Hyperolius mitchelli (HM772447) | Hyperolius cinnamomeoventris (FJ594108) | Hyperolius tuberilinguis (DQ284381) |  | Hyperolius puncticulatus (HM772967) | Hyperolius viridiflavus (AY323769) | Hyperolius alticola (DQ283902) | Hyperolius tuberilinguis (DQ282858) | Hyperolius puncticulatus (DQ282997 ) | Hyperolius tuberilinguis (DQ283399) | Hyperolius adspersus (JQ513640) | Hyperolius thomensis (DQ283600) |
| *Tachycnemis* |  |  | Tachycnemis seychellensis (AY341739) | Tachycnemis seychellensis (DQ284424) |  |  | Tachycnemis seychellensis (JQ073264) | Tachycnemis seychellensis (DQ284041) | Tachycnemis seychellensis (DQ282896) | Tachycnemis seychellensis (DQ283029) | Tachycnemis seychellensis (DQ283451) | Tachycnemis seychellensis (DQ283451) | Tachycnemis seychellensis (DQ283754) |
| *Kassina* |  |  | Kassina senegalensis (AF215495) | Kassina senegalensis (DQ284413) |  |  |  |  | Kassina senegalensis (DQ282891) | Kassina senegalensis (DQ283026) | Kassina senegalensis (DQ283437) | Kassina senegalensis (DQ283437) | Kassina senegalensis (DQ283746) |
| *Physalaemus* | Physalaemus cuvieri (AY819181) |  | Physalaemus cuvieri (AY843975) |  | Physalaemus cuvieri (AY819477) | Physalaemus cuvieri (AY819096) | Physalaemus cuvieri (AY844499) | Physalaemus gracilis (DQ284022) | Physalaemus cuvieri (AY844922) |  | Physalaemus gracilis (DQ283417) | Physalaemus gracilis (DQ283417) | Physalaemus cuvieri (AY844330) |
| *Pleurodema* | Pleurodema marmoratum (GQ345160) | Pleurodema marmoratum (GQ345174) | Pleurodema brachyops (AY843979) | Pleurodema brachyops (DQ284111) |  | Pleurodema marmoratum (GQ345273) | Pleurodema brachyops (AY844503) | Pleurodema brachyops (AY844721) | Pleurodema brachyops (AY844926) |  | Pleurodema thaul (JX442301) | Pleurodema thaul (JX442301) | Pleurodema marmoratum (GQ345144) |
| *Leptodactylus* | Leptodactylus didymus (AY819180) | Leptodactylus didymus (AY819259) | Leptodactylus albilabris (EF091402) | Leptodactylus discodactylus (DQ284410) | Leptodactylus mystacinus (AY911285) | Leptodactylus latrans (DQ158259) | Leptodactylus fuscus (AY323770) | Leptodactylus fuscus (AY323746) | Leptodactylus fuscus (DQ282862) | Leptodactylus melanonotus (DQ347193) | Leptodactylus latrans (DQ158417) | Leptodactylus latrans (DQ158417) | Leptodactylus fuscus (DQ283716) |
| *Paratelmatobius* |  |  |  | Paratelmatobius sp. (DQ284148) |  |  |  | Paratelmatobius sp. (DQ283814) | Paratelmatobius sp. (DQ282676) | Paratelmatobius sp. (DQ282925) | Paratelmatobius sp. (DQ283098) | Paratelmatobius sp. (DQ283098) | Paratelmatobius sp. (DQ283499) |
| *Scythrophrys* |  |  |  | Scythrophrys sawayae (DQ284149) |  |  |  |  |  | Scythrophrys sawayae (DQ282926) | Scythrophrys sawayae (DQ283099) | Scythrophrys sawayae (DQ283099) | Scythrophrys sawayae (DQ283500) |
| *Adelotus* |  |  |  | Adelotus brevis (DQ284307) |  |  |  | Adelotus brevis (DQ283948) | Adelotus brevis (DQ282800) |  | Adelotus brevis (DQ283298) | Adelotus brevis (DQ283297) | Adelotus brevis (DQ283638) |
| *Heleioporus* |  |  |  | Heleioporus australiacus (DQ284311) |  |  |  | Heleioporus australiacus (DQ283953) | Heleioporus australiacus (DQ282804) |  | Heleioporus australiacus (DQ283307) | Heleioporus australiacus (DQ283306) | Heleioporus australiacus (DQ283642) |
| *Lechriodus* |  |  |  | Lechriodus fletcheri (DQ284299) |  |  | Lechriodus melanopyga (AY583341) | Lechriodus fletcheri (DQ283942) | Lechriodus fletcheri (DQ282793) |  |  | Lechriodus melanopyga (DQ872915) | Lechriodus fletcheri (DQ283632) |
| *Limnodynastes* | Limnodynastes tasmaniensis (GQ345161) | Limnodynastes tasmaniensis (GQ345175) | Limnodynastes tasmaniensis (GQ345209) | Limnodynastes dumerilii (DQ284301) | Limnodynastes salmini (AY523775) |  | Limnodynastes salmini (AY364219) | Limnodynastes salmini (AY364400) | Limnodynastes dumerilii (DQ282794) |  | Limnodynastes depressus (DQ283308) | Limnodynastes peronii (DQ283245) | Limnodynastes depressus (DQ283643) |
| *Neobatrachus* |  |  |  | Neobatrachus sudelli (DQ284123) |  |  |  | Neobatrachus sudelli (AY844691) | Neobatrachus sudelli (AY844900) |  | Neobatrachus pictus (DQ283290) | Neobatrachus pictus (DQ283291) | Neobatrachus sudelli (AY844307) |
| *Notaden* | Notaden bennettii (AY819184) |  |  | Notaden melanoscaphus (DQ284302) | Notaden bennettii (AY819480) | Notaden bennettii (AY819099) |  | Notaden melanoscaphus (DQ283945) | Notaden melanoscaphus (DQ282795) |  | Notaden melanoscaphus (DQ283287) | Notaden melanoscaphus (DQ283288) |  |
| *Philoria* |  |  |  |  |  |  |  | Philoria sphagnicolus (DQ283957) |  |  | Philoria sphagnicolus (DQ283313) | Philoria sphagnicolus (DQ283313) | Philoria sphagnicolus (DQ283646) |
| *Mantella* |  |  | Mantella madagascariensis (NC_007888) | Mantella nigricans (DQ284056) | Mantella madagascariensis (NC_007888) | Mantella madagascariensis (HM998971) | Mantella madagascariensis (DQ019500) | Mantella aurantiaca (DQ283766) | Mantella aurantiaca (DQ282651) | Mantella madagascariensis (AF249164) | Mantella madagascariensis (NC_007888) | Mantella madagascariensis (NC_007888) | Mantella aurantiaca (DQ283460) |
| *Aglyptodactylus* |  |  | Aglyptodactylus madagascariensis (JN132845) |  |  |  | Aglyptodactylus madagascariensis (DQ347233) | Aglyptodactylus madagascariensis (DQ283785) |  | Aglyptodactylus madagascariensis (DQ282906) | Aglyptodactylus madagascariensis (DQ346997) | Aglyptodactylus madagascariensis (DQ346997) | Aglyptodactylus madagascariensis (DQ283469) |
| *Micrixalus* |  |  | Micrixalus fuscus (AF249086) |  |  |  |  | Micrixalus borealis (DQ283909) | Micrixalus borealis (DQ282766) | Micrixalus kottigeharensis (AF249184) |  | Micrixalus borealis (DQ283235) | Micrixalus borealis (DQ283603) |
| *Gastrophryne* | Gastrophryne carolinensis (AY819183) | Gastrophryne carolinensis (AY819261) |  | Gastrophryne elegans (DQ284404) | Gastrophryne carolinensis (AY819479) | Gastrophryne carolinensis (AY819098) | Gastrophryne olivacea (AB611896) | Gastrophryne olivacea (DQ283932) | Gastrophryne olivacea (DQ282784) | Gastrophryne elegans (DQ283019 ) | Gastrophryne olivacea (DQ347055) | Gastrophryne olivacea (DQ347055) | Gastrophryne olivacea (DQ283624) |
| *Xenorhina* |  |  |  |  |  |  | Xenorhina obesa (EF018048) |  |  |  | Xenorhina varia (EU100219) | Xenorhina varia (EU100335) |  |
| *Platypelis* |  |  |  | Platypelis grandis (DQ284392) |  |  | Platypelis grandis (EF396101) |  | Platypelis grandis (DQ282868) | Platypelis grandis (DQ283007) | Platypelis grandis (DQ283410) | Platypelis grandis (DQ283410) | Platypelis grandis (DQ283721) |
| *Hoplophryne* |  |  | Hoplophryne rogersi (FJ998373) | Hoplophryne rogersi (DQ284398) |  |  | Hoplophryne rogersi (EF018050) |  | Hoplophryne rogersi (DQ282876) | Hoplophryne rogersi (DQ283015) | Hoplophryne rogersi (DQ283419) | Hoplophryne rogersi (DQ283419) | Hoplophryne rogersi (DQ283730) |
| *Microhyla* |  |  | Microhyla heymonsi (NC_006406) | Microhyla sp. (DQ284400) | Microhyla heymonsi (NC_006406) | Microhyla sp. (HM998967) | Microhyla ornata (AY364198) | Microhyla ornata (AY364383) | Microhyla heymonsi (DQ282845) | Microhyla heymonsi (DQ282992) | Microhyla heymonsi (NC_006406) | Microhyla heymonsi (NC_006406) | Microhyla heymonsi (DQ283697) |
| *Phrynomantis* |  |  | Phrynomantis bifasciatus (FJ998374) | Phrynomantis bifasciatus (DQ284193) |  |  | Phrynomantis bifasciatus (EF396100) | Phrynomantis microps (JF837041) | Phrynomantis bifasciatus (DQ282709) | Phrynomantis bifasciatus (DQ282940) | Phrynomantis bifasciatus (AY531856) | Phrynomantis bifasciatus (AY531833) | Phrynomantis bifasciatus (DQ283545) |
| *Scaphiophryne* |  |  |  | Scaphiophryne marmorata (DQ284391) | Scaphiophryne marmorata (AY523765) |  | Scaphiophryne marmorata (AY364205) | Scaphiophryne marmorata (AY364390) | Scaphiophryne marmorata (DQ282867) | Scaphiophryne marmorata (AY844175) | Scaphiophryne marmorata (AY843751) | Scaphiophryne marmorata (AY843751) | Scaphiophryne marmorata (DQ283720) |
| *Arenophryne* |  |  |  | Arenophryne sp. (DQ284322) |  |  |  | Arenophryne sp. (DQ283965) |  |  |  | Arenophryne sp. (DQ283326) | Arenophryne sp. (DQ283656) |
| *Assa* |  |  |  | Assa darlingtoni (DQ284300) |  |  |  | Assa darlingtoni (DQ283943) |  |  |  | Assa darlingtoni (DQ283284) |  |
| *Crinia* |  |  |  | Crinia signifera (DQ284226) |  |  | Crinia signifera (AY948939) | Crinia nimbus (DQ283949) | Crinia signifera (DQ282737) |  | Crinia signifera (EU443926) | Crinia nimbus (DQ283299) | Crinia nimbus (DQ283639) |
| *Geocrinia* |  |  |  | Geocrinia victoriana (DQ284306) |  |  |  | Geocrinia victoriana (DQ283947) | Geocrinia victoriana (DQ282799) | Geocrinia victoriana (DQ282965) | Geocrinia victoriana (DQ283295) | Geocrinia victoriana (DQ283296) | Geocrinia victoriana (DQ283637) |
| *Metacrinia* |  |  |  | Metacrinia nichollsi (DQ284305) |  |  |  | Metacrinia nichollsi (DQ283946) | Metacrinia nichollsi (DQ282798) |  | Metacrinia nichollsi (DQ283292) | Metacrinia nichollsi (DQ283293) | Metacrinia nichollsi (DQ283636) |
| *Mixophyes* |  |  | Mixophyes carbinensis (JN677525) | Mixophyes carbinensis (DQ284315) |  |  | Mixophyes balbus (AY948940) | Mixophyes carbinensis (DQ283958) |  |  | Mixophyes carbinensis (DQ283315) | Mixophyes schevilli (JN657684) |  |
| *Myobatrachus* |  |  |  | Myobatrachus gouldii (DQ284313) |  |  | Myobatrachus gouldii (AY364226) | Myobatrachus gouldii (DQ283955) |  |  | Myobatrachus gouldii (DQ283309) | Myobatrachus gouldii (DQ283310) | Myobatrachus gouldii (DQ283644) |
| *Paracrinia* |  |  |  | Paracrinia haswelli (DQ284310) |  |  |  | Paracrinia haswelli (DQ283952) |  |  | Paracrinia haswelli (DQ283304) | Paracrinia haswelli (DQ283305) | Paracrinia haswelli (DQ283641) |
| *Pseudophryne* |  |  | Pseudophryne bibroni (AY843988) | Pseudophryne coriacea (DQ284314) |  |  |  | Pseudophryne coriacea (DQ283956) | Pseudophryne coriacea (DQ282806) |  | Pseudophryne coriacea (DQ283312) | Pseudophryne coriacea (DQ283311) | Pseudophryne coriacea (DQ283645) |
| *Rheobatrachus* |  |  |  | Rheobatrachus silus (DQ284295) |  |  | Rheobatrachus silus (AY948938) | Rheobatrachus silus (DQ283938) | Rheobatrachus silus (DQ282789) |  | Rheobatrachus silus (DQ283276) | Rheobatrachus silus (DQ283275) |  |
| *Spicospina* |  |  |  | Spicospina flammocaerulea (DQ284308) |  |  |  | Spicospina flammocaerulea (DQ283950) | Spicospina flammocaerulea (DQ282802) | Spicospina flammocaerulea (DQ282966) | Spicospina flammocaerulea (DQ283301) | Spicospina flammocaerulea (JF263329) | Spicospina flammocaerulea (DQ283640) |
| *Taudactylus* |  |  |  | Taudactylus acutirostris (DQ284296) |  |  |  | Taudactylus acutirostris (DQ283939) | Taudactylus acutirostris (DQ282790) |  | Taudactylus acutirostris (DQ283278) | Taudactylus acutirostris (DQ283277) |  |
| *Uperoleia* | Uperoleia littlejohni (AY819185) | Uperoleia littlejohni (AY819262) |  | Uperoleia laevigata (DQ284251) | Uperoleia littlejohni (AY819481) | Uperoleia littlejohni (AY819100) |  | Uperoleia laevigata (DQ283898) | Uperoleia laevigata (DQ282758) |  | Uperoleia laevigata (DQ283221) | Uperoleia talpa (JF263438) |  |
| *Nasikabatrachus* |  |  | Nasikabatrachus sahyadrensis (GU136138) |  |  |  | Nasikabatrachus sahyadrensis (AY364225) | Nasikabatrachus sahyadrensis (AY364406) |  |  | Nasikabatrachus sahyadrensis (GU143819) | Nasikabatrachus sahyadrensis (GU136108) |  |
| *Lankanectes* |  |  | Lankanectes corrugatus (AF249091) |  |  |  | Lankanectes corrugatus (GQ204608) | Lankanectes corrugatus (DQ019562) |  | Lankanectes corrugatus (AF249178) | Lankanectes corrugatus (DQ346971) | Lankanectes corrugatus (DQ346971) |  |
| *Nyctibatrachus* |  |  | Nyctibatrachus cf. aliciae (AF249073) |  | Nyctibatrachus aliciae (JF274075) |  | Nyctibatrachus cf. aliciae (DQ347210) | Nyctibatrachus cf. aliciae (AF249114) |  | Nyctibatrachus aliciae (AF249177) | Nyctibatrachus cf. aliciae (DQ346969) | Nyctibatrachus aliciae (JN644893) |  |
| *Macrogenioglottus* |  |  | Macrogenioglottus alipioi (FJ685665) |  |  |  | Macrogenioglottus alipioi (FJ685704) |  |  |  |  | Macrogenioglottus alipioi (FJ685684) |  |
| *Odontophrynus* |  |  | Odontophrynus carvalhoi (FJ685667) | Odontophrynus achalensis (DQ284273) | Odontophrynus occidentalis (AY948757) |  | Odontophrynus occidentalis (AY948934) | Odontophrynus americanus (AY844695) | Odontophrynus americanus (AY844901) |  | Odontophrynus achalensis (DQ283247) | Odontophrynus achalensis (DQ283247) | Odontophrynus achalensis (DQ283611) |
| *Proceratophrys* | Proceratophrys melanopogon (GQ345157) | Proceratophrys melanopogon (GQ345171) | Proceratophrys boiei (JN814580) | Proceratophrys avelinoi (DQ284065) |  | Proceratophrys melanopogon (GQ345270) | Proceratophrys boiei (JN814665) | Proceratophrys avelinoi (DQ283769) |  | Proceratophrys avelinoi (DQ282903) | Proceratophrys avelinoi (DQ283038) | Proceratophrys avelinoi (DQ283038) |  |
| *Petropedetes* |  |  | Petropedetes yakusini (JX546982) | Petropedetes yakusini (DQ284396) |  |  | Petropedetes parkeri (AY364213) | Petropedetes newtoni (DQ283869) | Petropedetes newtoni (DQ282727) | Petropedetes cf. parkeri (DQ347147) | Petropedetes yakusini (JX546942) | Petropedetes yakusini (JX546964) | Petropedetes yakusini (DQ283725) |
| *Phrynobatrachus* |  |  |  | Phrynobatrachus natalensis (DQ284395) |  |  | Phrynobatrachus natalensis (DQ347242) | Phrynobatrachus natalensis (DQ284019) | Phrynobatrachus natalensis (DQ282871) | Phrynobatrachus natalensis (DQ347145) | Phrynobatrachus natalensis (DQ283414) | Phrynobatrachus natalensis (DQ283414) | Phrynobatrachus natalensis (DQ283724) |
| *Ptychadena* |  |  | Ptychadena mascareniensis (AY341734) | Ptychadena mascareniensis (DQ284052) |  |  | Ptychadena mascareniensis (JQ073267) | Ptychadena mascareniensis (DQ283760) |  | Ptychadena mascareniensis (DQ282899) | Ptychadena mascareniensis (DQ022339) | Ptychadena mascareniensis (JF903872) |  |
| *Hildebrandtia* |  |  |  |  |  |  |  |  |  |  | Hildebrandtia ornata (AF261243) | Hildebrandtia ornata (AF215402) |  |
| *Amietia* |  |  |  | Amietia vertebralis (DQ284383) |  |  | Amietia vertebralis (FJ411454) |  | Amietia vertebralis (DQ282860) |  | Amietia vertebralis (DQ283402) | Amietia vertebralis (DQ283402) | Amietia vertebralis (DQ283714) |
| *Aubria* |  |  |  | Aubria subsigillata (DQ284341) |  |  |  | Aubria subsigillata (DQ283977) | Aubria subsigillata (DQ282724) | Aubria subsigillata (DQ282975) | Aubria subsigillata (DQ283350) | Aubria subsigillata (DQ283351) | Aubria subsigillata (DQ283675) |
| *Rana* |  |  | Rana japonica (AF077395) | Rana japonica (DQ284177) | Rana japonica (AY607313) | Rana japonica (AB728250) | Rana japonica (AB728272) | Rana japonica (AB728289) | Rana japonica (DQ282692) | Rana japonica (AB728323) | Rana japonica (DQ283136) | Rana japonica (DQ283136) | Rana japonica (DQ283529) |
| *Lithobates* |  |  | Rana clamitans (DQ792684) | Rana palmipes (DQ284369) |  |  | Rana palmipes (DQ347263) | Rana palmipes (DQ284001) | Rana palmipes (DQ282847) | Rana palmipes (DQ282994) | Rana palmipes (DQ347037) | Rana palmipes (DQ347037) | Rana palmipes (DQ283699) |
| *Indirana* |  |  | Indirana sp. (AF249080) |  |  |  | Indirana cf. beddomii (JQ596753) | Indirana cf. beddomii (JQ596795) |  | Indirana sp. (AY322338) | Indirana cf. beddomii (JQ596729) | Indirana cf. beddomii (JQ596677) |  |
| *Rhacophorus* |  |  | Rhacophorus schlegelii (NC_007178) | Rhacophorus calcaneus (DQ284366) | Rhacophorus schlegelii (NC_007178) | Rhacophorus norhayatii (AB728248) | Rhacophorus norhayatii (AB728270) | Rhacophorus norhayatii (AB728287) | Rhacophorus calcaneus (DQ282843) | Rhacophorus norhayatii (AB728321) | Rhacophorus schlegelii (NC_007178) | Rhacophorus schlegelii (NC_007178) | Rhacophorus calcaneus (DQ283695) |
| *Theloderma* |  |  | Theloderma asperum (GQ204542) | Theloderma corticale (DQ284080) |  | Theloderma asperum (GQ285728) | Theloderma asperum (GQ285760) | Theloderma corticale (DQ283779) | Theloderma corticale (DQ282659) | Theloderma corticale (DQ282904) | Theloderma corticale (DQ283050) | Theloderma corticale (DQ283050) | Theloderma rhododiscus (DQ283706) |
| *Insuetophrynus* |  |  | Insuetophrynus acarpicus (AY386400) |  |  |  |  |  |  |  | Insuetophrynus acarpicus (JX204223) | Insuetophrynus acarpicus (JX204223) |  |
| *Rhinoderma* |  |  | Rhinoderma darwinii (DQ502589) | Rhinoderma darwinii (DQ284320) | Rhinoderma darwinii (AY523783) |  | Rhinoderma darwinii (AY364222) | Rhinoderma darwinii (DQ283963) | Rhinoderma darwinii (DQ282813) |  | Rhinoderma darwinii (DQ283324) | Rhinoderma darwinii (DQ283324) | Rhinoderma darwinii (DQ283654) |
| *Sechellophryne* |  |  |  |  |  |  | Sechellophryne gardineri (DQ872923) |  |  |  |  | Sechellophryne gardineri (HQ625494) |  |
| *Sooglossus* |  |  | Sooglossus thomasseti (AY341742) | Sooglossus sechellensis (DQ284423) |  |  | Sooglossus sechellensis (DQ872921) | Sooglossus sechellensis (DQ284040) | Sooglossus sechellensis (DQ282895) | Sooglossus thomasseti (DQ347167) | Sooglossus thomasseti (AY341632) | Sooglossus sechellensis (JF784383) | Sooglossus sechellensis (DQ283753) |
| *Phrynopus* | Phrynopus bracki (GQ345150) | Phrynopus bracki (GQ345168) | Phrynopus bracki (GQ345202) | Phrynopus sp. (DQ284371) |  | Phrynopus bracki (GQ345263) | Phrynopus bracki (GQ345281) | Phrynopus bracki (GQ345303) | Phrynopus bracki (GQ345314) | Phrynopus bracki (EF493507) | Phrynopus bracki (EF493709) | Phrynopus bracki (EF493709) | Phrynopus sp. (AY844323) |
| *Strabomantis* | Strabomantis biporcatus (GQ345152) |  | Strabomantis biporcatus (GQ345204) | Strabomantis bufoniformis (DQ284203) | Strabomantis biporcatus (GQ345249) | Strabomantis biporcatus (GQ345265) | Strabomantis biporcatus (GQ345283) |  | Strabomantis bufoniformis (DQ282718) | Strabomantis bufoniformis (DQ282942) | Strabomantis bufoniformis (DQ283165) | Strabomantis bufoniformis (DQ283165) | Strabomantis bufoniformis (DQ283555) |
| *Telmatobius* | Telmatobius truebae (AY819182) | Telmatobius truebae (AY819260) | Telmatobius sibiricus (GU060615) | Telmatobius marmoratus (DQ284068) |  | Telmatobius truebae (AY819097) | Telmatobius bolivianus (AY583344) | Telmatobius verrucosus (DQ283770) | Telmatobius bolivianus (AY844952) | Telmatobius sp. (DQ347182) | Telmatobius verrucosus (DQ283040) | Telmatobius verrucosus (DQ283040) | Telmatobius bolivianus (AY844355) |

Blotto BL, Nuñez JJ, Basso NSG, Úbeda CA, Wheeler WC, et al. (2013). Phylogenetic relationships of a Patagonian frog radiation, the *Alsodes* + *Eupsophus* clade (Anura: Alsodidae), with comments on the supposed paraphyly of *Eupsophus*. Cladistics 29(2): 113–31.

Fouquet A, Blotto BL, Maronna MM, Verdade VK, Juncá FA, et al. **2013**. Unexpected phylogenetic positions of the genera *Rupirana* and *Crossodactylodes* reveal insights into the biogeography and reproductive evolution of leptodactylid frogs. **Mol Phylogenet Evol** 67: 445–457.

Faivovich J, Nicoli L, Blotto BL, Pereyra MO, Baldo D, et al. (2014) Big, bad, and beautiful: Phylogenetic relationships of the horned frogs (Anura: Ceratophryidae). South Am J Herpetol 9:(3) 207-27
