# Supplementary material for: The Gondwana Breakup and the History of the Atlantic and Indian Oceans Unveils Two New Clades for Early Neobatrachian Diversification
Source: PLoS One. 2015 Nov 30;10(11):e0143926. doi: 10.1371/journal.pone.0143926 (PMC4664409; doi:10.1371/journal.pone.0143926)
Supplement: S2 Table — Geographical distribution and taxonomic information for each species analysed. (DOC) [file pone.0143926.s007.doc]

Table S2: Table with taxonomic information and biogeographical distribution used for biogeographical analysis. Numbers into parentheses represent the total number of species that contained in taxa. *i.s*.: *insert sedis*. AT, Afrotropical: all territory of African continent plus Middle East; AU, Australian: Australian region and adjacent islands; MA, Madagascan: Madagascar and adjacent islands, including Seychelles, Mauritius and Reunion; NA, Nearctic: North America, except South Mexico and northern end of Canada and Greenland; NT, Neotropical: South America; OC, Oceanian: Papua New Guinea and adjacent islands; OR, Oriental: Southeast Asia plus Bangladesh, Bhutan, Nepal and Pakistan; PA, Palearctic: all European and Asian countries, except Southern of China, Southeast Asia, India, Bangladesh, Bhutan, Japan, Nepal, Pakistan and Sri Lanka; PM, Panamanian: Central America and adjacent islands; S-A, Saharo-Arabian: northern Africa, except Sudan, plus Middle East; SJ, Sino-Japanese: Tibetan region, southern China and Japan; IN: India and Sri Lanka. *Distribution followed biogeographical regions of Holt *et al.* (2013), except abbreviation “IN”.

| **Clades *sensu* Frost *et al.*(2006)** | | | | **Family** | **Subfamily** | **Genus** | **Distribution*** |
| --- | --- | --- | --- | --- | --- | --- | --- |
| Archaeobatrachia |  |  |  | Bombinatoridae (8) |  | *Bombina* (6) | PA, OR, SJ |
|  |  |  |  | Pelobatidae (4) |  | *Pelobates* (4) | PA, S-A |
|  |  |  |  | Pipidae (33) |  | *Pipa* (4) | NT, PM |
|  |  |  |  |  |  | *Xenopus* (21) | AT |
| Neobatrachia (5965) | *i.s*. (7) |  |  | Heleophrynidae (7) |  | *Hadromophryne* (1) | AT |
|  |  |  |  |  |  | *Heleophryne* (6) | AT |
|  | Hyloides (3593) |  |  | Nasikabatrachidae (1) |  | *Nasikabatrachus* (1) | IN |
|  |  |  |  | Sooglossidae (4) |  | *Sechellophryne* (2) | MA |
|  |  |  |  |  |  | *Sooglossus* (2) | MA |
|  |  |  | Australobatrachia (136) | Calyptocephalellidae (5) |  | *Calyptocephalella* (1) | NT |
|  |  |  |  |  |  | *Telmatobufo* (4) | NT |
|  |  |  |  | Limnodynastidae (43) |  | *Adelotus* (1) | AU |
|  |  |  |  |  |  | *Heleioporus* (6) | AU |
|  |  |  |  |  |  | *Lechriodus* (4) | AU, OC |
|  |  |  |  |  |  | *Limnodynastes* (11) | AU, OC |
|  |  |  |  |  |  | *Neobatrachus* (9) | AU |
|  |  |  |  |  |  | *Notaden* (4) | AU |
|  |  |  |  |  |  | *Philoria* (6) | AU |
|  |  |  |  | Myobatrachidae (88) |  | *Arenophryne* (2) | AU |
|  |  |  |  |  |  | *Assa* (1) | AU |
|  |  |  |  |  |  | *Crinia* (17) | AU |
|  |  |  |  |  |  | *Geocrinia* (7) | AU |
|  |  |  |  |  |  | *Metacrinia* (1) | AU |
|  |  |  |  |  |  | *Mixophyes* (8) | AU |
|  |  |  |  |  |  | *Myobatrachus* (1) | AU |
|  |  |  |  |  |  | *Paracrinia* (1) | AU |
|  |  |  |  |  |  | *Pseudophryne* (14) | AU |
|  |  |  |  |  |  | *Rheobatrachus* (2) | AU |
|  |  |  |  |  |  | *Spicospina* (1) | AU |
|  |  |  |  |  |  | *Taudactylus* (6) | AU |
|  |  |  |  |  |  | *Uperoleia* (27) | AU, OC |
|  |  |  | Nobleobatrachia (3452) | Allophrynidae (2) |  | *Allophryne* (2) | NT |
|  |  |  |  | Alsodidae (30) |  | *Alsodes* (19) | NT |
|  |  |  |  |  |  | *Eupsophus* (10) | NT |
|  |  |  |  |  |  | *Limnomedusa* (1) | NT |
|  |  |  |  | Aromobatidae (115) | Allobatinae (46) | *Allobates* (46) | NT |
|  |  |  |  |  | Aromobatinae (37) | *Aromobates* (18) | NT |
|  |  |  |  | Batrachylidae (14) |  | *Atelognathus* (7) | NT |
|  |  |  |  |  |  | *Batrachyla* (5) | NT |
|  |  |  |  |  |  | *Hylorina* (1) | NT |
|  |  |  |  | Brachycephalidae (52) |  | *Brachycephalus* (20) | NT |
|  |  |  |  |  |  | *Ischnocnema* (32) | NT |
|  |  |  |  | Bufonidae (571) |  | *Adenomus* (3) | IN |
|  |  |  |  |  |  | *Amietophrynus* (40) | AT, S-A |
|  |  |  |  |  |  | *Anaxyrus* (22) | NA |
|  |  |  |  |  |  | *Ansonia* (26) | NA |
|  |  |  |  |  |  | *Atelopus* (96) | NT, PM |
|  |  |  |  |  |  | *Bufo* (17) | PA, OR, SJ, S-A |
|  |  |  |  |  |  | *Bufotes* (14) | PA, S-A, SJ |
|  |  |  |  |  |  | *Capensibufo* (2) | AT |
|  |  |  |  |  |  | *Churamiti* (1) | AT |
|  |  |  |  |  |  | *Amazophrynella* (6) | NT |
|  |  |  |  |  |  | *Didynamipus* (1) | AT |
|  |  |  |  |  |  | *Duttaphrynus* (28) | IN, S-A, OR, SJ |
|  |  |  |  |  |  | *Epidalea* (1) | PA |
|  |  |  |  |  |  | *Ghatophryne* (2) | IN |
|  |  |  |  |  |  | *Incilius* (38) | NA, NT, PM |
|  |  |  |  |  |  | *Ingerophrynus* (12) | OR |
|  |  |  |  |  |  | *Leptophryne* (2) | OR |
|  |  |  |  |  |  | *Melanophryniscus* (26) | NT |
|  |  |  |  |  |  | *Mertensophryne* (14) | AT |
|  |  |  |  |  |  | *Nannophryne* (4) | NT |
|  |  |  |  |  |  | *Nectophryne* (2) | AT |
|  |  |  |  |  |  | *Nectophrynoides* (13) | AT |
|  |  |  |  |  |  | *Nimbaphrynoides* (1) | AT |
|  |  |  |  |  |  | *Oreophrynella* (9) | NT |
|  |  |  |  |  |  | *Osornophryne* (11) | NT |
|  |  |  |  |  |  | *Pedostibes* (5) | IN, OR |
|  |  |  |  |  |  | *Pelophryne* (11) | OR, SJ |
|  |  |  |  |  |  | *Peltophryne* (12) | PM |
|  |  |  |  |  |  | *Phrynoidis* (2) | OR |
|  |  |  |  |  |  | *Poyntonophrynus* (10) | AT |
|  |  |  |  |  |  | *Rhaebo* (10) | PM, NT |
|  |  |  |  |  |  | *Rhinella* (86) | NT, NA, PM |
|  |  |  |  |  |  | *Schismaderma* (1) | AT |
|  |  |  |  |  |  | *Vandijkophrynus* (5) | AT |
|  |  |  |  |  |  | *Werneria* (6) | AT |
|  |  |  |  |  |  | *Wolterstorffina* (3) | AT |
|  |  |  |  | Centrolenidae (149) | Hyalinobatrachinae (30) | *Hyalinobatrachium* (28) | NT |
|  |  |  |  |  | Centroleninae (118) | *Nymphargus* (35) | NT |
|  |  |  |  | Ceratophryidae (12) |  | *Ceratophrys* (8) | NT |
|  |  |  |  |  |  | *Chacophrys* (1) | NT |
|  |  |  |  |  |  | *Lepidobatrachus* (3) | NT |
|  |  |  |  | Ceuthomantidae (4) |  | *Ceuthomantis* (4) | NT |
|  |  |  |  | Craugastoridae (713) | Craugastorinae (115) | *Craugastor* (113) | NA, NT, PM |
|  |  |  |  |  |  | *Haddadus* (2) | NT |
|  |  |  |  |  | Pristimantinae (509) | *Phrynopus* (25) | NT |
|  |  |  |  |  | Strabomantinae (22) | *Strabomantis* (17) | NT, PM |
|  |  |  |  | Cycloramphidae (40) |  | *Cycloramphus* (28) | NT |
|  |  |  |  |  |  | *Thoropa* (6) | NT |
|  |  |  |  |  |  | *Zachaenus* (2) | NT |
|  |  |  |  | Dendrobatidae (177) | Dendrobatinae (54) | *Dendrobates* (5) | NT, PM |
|  |  |  |  |  | Colostethinae (62) | *Silverstoneia* (3) | NT, PM |
|  |  |  |  | Eleutherodactylidae (205) | Phyzelaphryninae (8) | *Adelophryne* (7) | NT |
|  |  |  |  |  | Eleutherodactylinae (197) | *Eleutherodactylus* (186) | PM |
|  |  |  |  | Hemiphractidae (100) |  | *Flectonotus* (2) | NT |
|  |  |  |  |  |  | *Hemiphractus* (6) | NT, PM |
|  |  |  |  | Hylidae (926) | Pelodryadinae (201) | *Litoria* (201) | AU |
|  |  |  |  |  | Phyllomedusinae (58) | *Phyllomedusa* (30) | NT, PM |
|  |  |  |  |  | Hylinae (667) | *Scinax* (111) | NT, PM |
|  |  |  |  | Hylodidae (42) |  | *Crossodactylus* (11) | NT |
|  |  |  |  |  |  | *Hylodes* (24) | NT |
|  |  |  |  | Leptodactylidae (189) | Leptodactylinae (94) | *Leptodactylus* (75) | NA, NT, PM |
|  |  |  |  |  | Leiuperinae (88) | *Physalaemus* (45) | NT |
|  |  |  |  |  | Leiuperinae (88) | *Pleurodema* (15) | NT |
|  |  |  |  |  | Paratelmatobiinae (7) | *Paratelmatobius* (6) | NT |
|  |  |  |  |  | Paratelmatobiinae (7) | *Scythrophrys* (1) | NT |
|  |  |  |  | Odontophrynidae (40) |  | *Macrogenioglottus* (1) | NT |
|  |  |  |  |  |  | *Odontophrynus* (12) | NT |
|  |  |  |  |  |  | *Proceratophrys* (27) | NT |
|  |  |  |  | Rhinodermatidae (3) |  | *Insuetophrynus* (1) | NT |
|  |  |  |  |  |  | *Rhinoderma* (2) | NT |
|  |  |  |  | Telmatobiidae (61) |  | *Telmatobius* (61) | NT |
|  | Ranoides (2372) | Allopadanura (906) |  | Microhylidae (519) | Gastrophryninae (54) | *Gastrophryne*(4) | NA, PM |
|  |  |  |  |  | Asterophryinae (274) | *Xenorhina* (31) | OC |
|  |  |  |  |  | Cophylinae (59) | *Platypelis* (12) | MA |
|  |  |  |  |  | Hoplophryninae (3) | *Hoplophryne* (2) | AT |
|  |  |  |  |  | Microhylinae (71) | *Microhyla* (31) | IN, SJ, OR |
|  |  |  |  |  | Phrynomerinae (5) | *Phrynomantis* (5) | AT |
|  |  |  |  |  | Scaphiophryninae (10) | *Scaphiophryne* (8) | MA |
|  |  |  | Afrobatrachia (387) | Hyperoliidae (210) |  | *Hyperolius* (131) | AT |
|  |  |  |  |  |  | *Tachycnemis* (1) | MA |
|  |  |  |  |  |  | *Kassina* (16) | AT |
|  |  |  |  | Arthroleptidae (144) | Astyosterninae (30) | *Scotobleps* (1) | AT |
|  |  |  |  |  | Arthroleptinae (63) | *Arthroleptis* (47) | AT |
|  |  |  |  | Brevicipitidae (33) |  | *Breviceps* (16) | AT |
|  |  |  |  |  |  | *Callulina* (9) | AT |
|  |  | Natatanura (1466) |  | Ceratobatrachidae (85) |  | *Ceratobatrachus* (1) | OC |
|  |  |  |  |  |  | *Batrachylodes* (8) | OC |
|  |  |  |  | Conrauidae (6) |  | *Conraua* (6) | AT |
|  |  |  |  | Dicroglossidae (181) | Dicroglossinae (157) | *Limnonectes* (56) | SJ, OC, OR |
|  |  |  |  |  |  | *Nannophrys* (4) | IN |
|  |  |  |  | Mantellidae (204) | Mantellinae (125) | *Mantella* (16) | MA |
|  |  |  |  |  | Laliostominae (4) | *Aglyptodactylus* (3) | MA |
|  |  |  |  | Micrixalidae (11) |  | *Micrixalus* (11) | IN |
|  |  |  |  | Nyctibatrachidae (28) |  | *Lankanectes* (1) | IN |
|  |  |  |  |  |  | *Nyctibatrachus* (27) | IN |
|  |  |  |  | Petropedetidae (12) |  | *Petropedetes* (12) | AT |
|  |  |  |  | Phrynobatrachidae (85) |  | *Phrynobatrachus* (85) | AT |
|  |  |  |  | Ptychadenidae (53) |  | *Ptychadena* (49) | AT |
|  |  |  |  |  |  | *Hildebrandtia* (3) | AT |
|  |  |  |  | Pyxicephalidae (72) | Cacosterninae (66) | *Amietia* (15) | AT |
|  |  |  |  |  | Pyxicephalinae (6) | *Aubria* (2) | AT |
|  |  |  |  | Ranidae (355) |  | *Rana* (48) | PA, NA, OR, SJ |
|  |  |  |  |  |  | *Lithobates* (49) | NT |
|  |  |  |  | Ranixalidae (10) |  | *Indirana* (10) | IN |
|  |  |  |  | Rhacophoridae (345) | Rhacophorinae (341) | *Rhacophorus* (81) | IN, OR, SJ |
|  |  |  |  |  |  | *Theloderma* (22) | IN, OR |
